# A Lightweight Identity-Based Secure Communication Scheme for In-Vehicle Networks

**DOI:** 10.3390/s26216740

**Published:** 2026-10-24

**Authors:** Annan Li, Yuanfang Wang, Zhijiao Bai, Zhenghua Qian, Ying Chen, Mu Han, Shidian Ma, Hai Wang

**Affiliations:** 1 Yangzhou Yaxing Motor Coach Co., Ltd., Yangzhou 225116, Chinaqianzhenghua@asiastarbus.com (Z.Q.); chenying02@asiastarbus.com (Y.C.); 2 School of Computer Science and Communication Engineering, Jiangsu University, Zhenjiang 212013, China; 2222408026@stmail.ujs.edu.cn (Y.W.);; 3 Automotive Engineering Research Institute, Jiangsu University, Zhenjiang 212013, China; 4 School of Automotive and Traffic Engineering, Jiangsu University, Zhenjiang 212013, China

**Keywords:** in-vehicle networks, identity-based cryptography, authenticated key agreement, lightweight secure communication, session key management

## Abstract

The increasing connectivity of intelligent vehicles exposes in-vehicle networks to message tampering, replay, impersonation, and unauthorized access. This paper presents an identity-based secure communication scheme that separates authenticated session establishment from established-session data protection. A trusted Key Generation Center provisions identity-bound private keys offline. The T-Box and a target ECU then perform mutual authentication, derive a shared session root key, and complete explicit bidirectional key confirmation. Direction- and purpose-specific subkeys are used with SM4-CTR and HMAC-SM3 to protect subsequent traffic. In the ProVerif symbolic model, all eleven secrecy and injective-correspondence queries return true under the stated assumptions. On the Windows/Python 3.12.7/UDP prototype, the median single-ECU handshake time is 287.560 ms, and the paired sender-plus-receiver processing median is 1.036 ms per 8-byte message. Under the stated unstuffed 500-kbit/s classical-CAN model, the selected profile requires 138.75 bits per message and yields 49.95% offered load for 18 ECUs at 100 messages/s per ECU. All 1200 frame-mutation trials were rejected without unauthorized plaintext release. These results characterize the symbolic model, host implementation, and analytical traffic model; they do not constitute target-ECU or physical-bus measurements.

## 1. Introduction

Modern vehicle electronic and electrical architectures use CAN/CAN FD, Automotive Ethernet, Unified Diagnostic Services (UDS), and Diagnostics over CAN (DoCAN) to support remote diagnostics, over-the-air software updates, parameter configuration, and vehicle management [1,2,3]. External connectivity also exposes in-vehicle communication to eavesdropping, tampering, replay, impersonation, and unauthorized access [4,5,6]. Because the Telematics Control Unit (T-Box) connects external services to the in-vehicle domain, compromise of this access path can enable diagnostic abuse, command injection, or software-update manipulation [7,8,9]. Accordingly, this study examines authenticated session establishment and protected communication between a T-Box and in-vehicle ECUs.

Secure in-vehicle communication commonly relies on symmetric mechanisms or certificate-based public-key infrastructures. Symmetric cryptography is suitable for frequent confidentiality and integrity processing but does not independently provide identity authentication and session-key establishment. Certificate-based schemes provide these functions but require certificate issuance, distribution, validation, renewal, and revocation over the vehicle life cycle. These life-cycle operations become more complex as ECU counts and vehicle service lives increase.

Identity-based cryptography derives a public key from an entity’s identity and therefore avoids per-node public-key certificates [10,11]. This approach transfers trust and life-cycle responsibilities to a Key Generation Center (KGC), which generates, provisions, renews, and revokes identity-bound private keys. Vehicle identifiers, functional addresses, ECU identifiers, and domain identifiers can be used as identity inputs when their uniqueness and provisioning procedures are controlled by the vehicle manufacturer or an authorized maintenance process.

Pairing-based authenticated key establishment is more computationally intensive than symmetric data protection. Because session establishment is less frequent than routine message transmission, the proposed organization confines identity-based operations to session setup and uses symmetric primitives after a session has been established.

This paper develops an identity-based secure communication scheme for resource-constrained in-vehicle networks. The protocol combines identity-bound authenticated session establishment, explicit key confirmation, directional subkey derivation, authenticated data protection, and isolated T-Box session contexts for multiple ECUs. It supports T-Box decrypt-and-forward via the gateway and end-to-end ciphertext-forwarding modes for off-board access.

A multi-process Windows/Python prototype measures session-establishment time, established-session processing cost, multi-ECU scaling, and rejection behavior under controlled frame mutation and loss. Classical-CAN communication cost and schedulability are evaluated analytically under the stated traffic model. The results characterize the host implementation and analytical traffic model; they do not measure target-ECU execution or physical-bus timing.

Vehicle security research also examines privacy-preserving tracking and localization, as well as quality of service and data dissemination in external vehicular networks. Recent studies consider privacy-preserving sensor selection for cloud-based vehicle tracking [12], output-mask fusion filtering for RIS-assisted vehicle localization [13], and VANET quality of service and data dissemination [14]. These studies underscore the need to protect vehicle data across sensing and communication domains. Because they address different layers from the intra-vehicle session establishment and protected CAN data plane studied here, they are not direct protocol baselines.

Groza and Murvay [15] investigated identity-based key exchange for in-vehicle networks, comparing signature-based and identity-based variants on automotive-grade controllers and in CANoe simulations for CAN-FD and FlexRay. This study integrates SM9-based low-frequency authenticated session establishment, transcript-bound explicit bidirectional key confirmation, direction- and purpose-separated subkeys, a protected high-frequency data plane, isolated T-Box security contexts for multiple ECUs, and T-Box decrypt-and-forward via the gateway and end-to-end ciphertext-forwarding modes in one framework.

The main contributions of this paper are summarized as follows:

(1) A layered identity-based secure communication framework is developed for resource-constrained in-vehicle networks. An SM9-based identity-bound session layer is used for offline provisioning and authenticated session establishment, while symmetric protection is used for established-session traffic. This separation reduces per-node certificate issuance, distribution, and online validation requirements without introducing a new identity-based cryptographic primitive.
(2) A T-Box-oriented multi-session architecture is developed with an isolated security context for each target ECU. It supports T-Box decrypt-and-forward via the gateway and end-to-end ciphertext-forwarding modes to isolate sessions and control off-board access across multiple ECUs.
(3) An authenticated session and data-plane mechanism combines ordered-transcript binding, explicit bidirectional key confirmation, direction- and purpose-specific subkeys, SM4-CTR encryption, HMAC-SM3 authentication, sequence-based freshness, and parameterized batch authentication with verify-before-release processing.
(4) The integrated design is evaluated using an eleven-query ProVerif symbolic model, adversarial frame-mutation tests, Windows/Python/UDP host measurements, and analytical classical-CAN load and schedulability models. The findings are limited to the stated symbolic, host software, and analytical settings and do not constitute validation on target ECUs or a physical CAN/CAN-FD bus.

The contribution is the system-level integration and evaluation of an SM9-based identity-bound protocol variant rather than a new identity-based primitive. Full forward secrecy after later compromise of a participating long-term identity key and validation on target ECUs or a physical CAN/CAN-FD bus are not claimed.

## 2. Related Work

Research on secure in-vehicle communication has moved beyond individual-message protection toward authentication, key agreement, and deployment constraints. Recent reviews describe work on intrusion detection, secure communication architectures, and key management [16,17,18,19]. Given the limited CAN payload, strict timing requirements, and ECU resource constraints, the literature can be grouped into data protection, authentication and key agreement, deployment adaptation, and the use of Chinese commercial cryptographic algorithms. In-vehicle networks commonly use CAN/CAN FD and Automotive Ethernet. Classical-CAN’s small payload means that many diagnostic and long-message exchanges rely on ISO-TP/UDS, so security designs must account for field overhead, segmentation, and latency [20,21,22,23]. ECU masquerade detection has also been studied using hybrid frame-interval and bus-voltage features [24].

(1) Lightweight security mechanisms for data protection

Early in-vehicle protection mechanisms used symmetric encryption, message authentication codes (MACs), and related constructions to provide confidentiality and integrity [16,22,23]. Their per-message processing cost is generally lower than that of public-key operations, making them relevant to resource-constrained ECUs. Prior CAN-authentication studies consequently examine full-length MACs, truncated MACs, and authentication formats designed for payload-constrained frames [22,23,25].

These mechanisms impose a payload–security trade-off. Longer authentication fields increase payload occupancy and segmentation pressure in CAN/CAN-FD messages, whereas truncation reduces the ideal forgery-resistance bound. Prior studies report corresponding increases in message length, bus demand, and processing latency when authentication fields or additional security messages are introduced [20,21,22,23,25].

(2) Cryptographic mechanisms for authentication and key agreement

Symmetric protection alone does not resolve how nodes establish identity trust and fresh session keys. Researchers have therefore introduced digital signatures, authentication protocols, and session key distribution mechanisms to provide mutual authentication and secure session establishment [16,26]. In recent years, research on CAN bus authentication mechanisms has expanded beyond message authenticity and integrity toward the coordinated design of multiple security objectives, including node identity confirmation, session initialization, key agreement, and replay protection [22,25,26,27]. Sender authentication with efficient key management is also considered in [28].

Digital-signature and certificate-based mechanisms support identity authentication and session establishment but add certificate and public-key life-cycle operations. In vehicles with many nodes and long service lives, issuance, distribution, validation, update, and revocation must be integrated with manufacturing and maintenance procedures [27]. Public-key operations also add computation, storage, and communication costs relative to symmetric per-message protection. Comparative studies therefore evaluate not only cryptographic properties but also deployment dependencies and life-cycle management [16,17,18].

(3) Engineering adaptation for vehicular scenarios

Research on CAN FD security increasingly addresses deployment within the protocol stack. Studies examine how to incorporate encryption and authentication into frame structures while accounting for message length, segmentation, bus load, and real-time constraints [20,21,23]. AUTOSAR SecOC provides an engineering reference for authenticity and freshness protection. Related work examines its authentication-field layout, freshness mechanisms, and implementation cost. SecOC appends authentication information to PDUs and uses freshness values to resist replay, but these functions consume software resources [25,26,29].

Latency studies analyze the worst-case response time of security-aware CAN FD and show that cryptographic overhead must be assessed alongside real-time requirements [20,23]. Accordingly, engineering designs often separate session establishment from data transfer: costly authentication and key agreement occur during initialization, followed by symmetric protection for routine messages [21,25,29].

Certificate-based public-key schemes provide established authentication and key-management procedures but require certificate life-cycle operations. Identity-based cryptography instead binds public-key derivation to an identity while relying on KGC-based private-key provisioning and revocation. In the proposed scheme, identity-based operations are used during session establishment, whereas established-session traffic uses symmetric encryption and authentication. This division follows the engineering separation between infrequent session setup and frequent data protection considered in prior vehicular security work [25,26,29].

Within the comparison scope of this paper, the reviewed studies do not jointly address identity binding, session-key agreement, data-plane protection, and secure T-Box off-board access under ECU resource constraints [16,17,18,25]. Symmetric-only approaches do not independently establish node identities and session keys; certificate-based approaches add life-cycle management; and many lightweight schemes focus on either authentication or data protection.

To address these gaps, this paper proposes a lightweight identity-based secure communication scheme for in-vehicle networks. The framework confines identity-based authenticated key agreement to session establishment and uses symmetric cryptography for established-session data. It avoids per-node certificate issuance, distribution, and online validation in the communication path while retaining KGC-based provisioning, revocation, and replacement responsibilities. Separating expensive session setup from high-frequency data protection is intended to reduce recurring processing overhead for resource-constrained in-vehicle nodes.

Groza and Murvay [15] compare signed, pairing-based identity-based, and pairing-free identity-based key-exchange variants for CAN-FD and FlexRay and evaluate automotive-controller computation and CANoe bandwidth. Relative to that work, the present scheme studies system-level integration of an SM9-based identity-bound session layer with transcript-bound bidirectional confirmation, direction- and purpose-specific subkeys, an authenticated data path, isolated per-ECU T-Box contexts, and two forwarding modes. The implementation is evaluated in a Windows/Python/UDP environment, so its host measurements are not treated as directly comparable to the automotive-controller or CANoe results in [15]. Table 1 summarizes the scope and reported deployment characteristics of representative prior work.

## 3. Identity-Based Secure Communication Scheme for In-Vehicle Nodes

### 3.1. System Model

The in-vehicle system comprises a T-Box, an in-vehicle gateway, and multiple Electronic Control Units (ECUs). The vehicle communicates with external services through cellular, Wi-Fi, or Bluetooth networks, while local diagnostic access is provided through the OBD-II interface. The T-Box serves as the main access point between external services and the in-vehicle network. It receives remote diagnostic, configuration, control, and OTA requests, identifies the target ECU, and manages the corresponding secure session. The in-vehicle gateway connects the T-Box to the internal CAN/CAN-FD network and forwards authorized messages to ECUs responsible for braking, body control, battery management, and powertrain functions.

Because ECUs have limited computing and storage resources, computationally intensive identity-based cryptographic operations are performed only during session establishment. Lightweight symmetric cryptographic operations are then used for subsequent data transmission. During system initialization, a trusted Key Generation Center (KGC) establishes the system parameters and generates an identity-based private key for each T-Box, gateway, and ECU according to its unique identity. These private keys are securely provisioned during vehicle manufacturing or maintenance. During operation, the T-Box performs mutual authentication and session key agreement with each target ECU through the gateway and maintains a separate session state for each ECU. The attack surface includes both remote network access and physical diagnostic access through the OBD-II interface. The model also covers secure message forwarding and session isolation across multiple T-Box–ECU sessions.

### 3.2. Threat Model and Security Requirements

In the proposed system model, a trusted Key Generation Center (KGC) initializes the system public parameters and generates an identity-based private key for each T-Box, gateway, and ECU according to its unique identity. The identity information of a node, such as its vehicle identifier, functional address, and ECU type, is used as its public-key identifier, thereby eliminating the need for conventional public-key certificates. The generated private keys are securely provisioned into the corresponding nodes during vehicle production or maintenance. The T-Box acts as the primary interface between external communication networks and the in-vehicle network, while the gateway controls and forwards messages between the external access domain and the ECUs connected through CAN or CAN FD. Each T-Box–ECU pair establishes an independent secure session for subsequent message exchange. The authenticated key agreement analyzed in Section 3.5 and Section 4 uses the T-Box and target ECU private keys; gateway authentication is not included in those protocol and verification claims.

**Figure 1:**
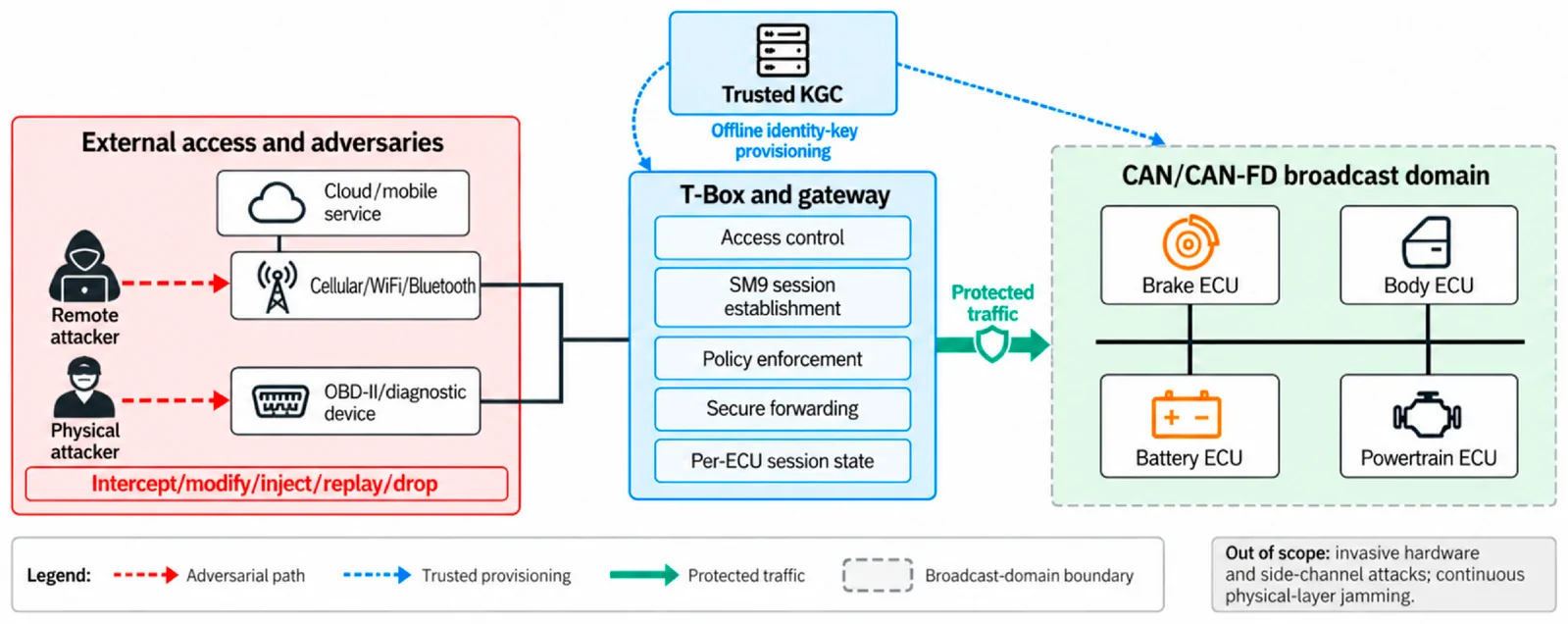
Threat model, trust boundary, adversarial paths, and protected in-vehicle communication domain.

Figure 1 summarizes the trust boundary and attack surface. The trusted KGC remains outside the adversarial domain and provisions identity-bound private keys through the offline channel. Remote attackers may access the system through cloud or wireless interfaces, while physical attackers may use the OBD-II diagnostic interface; both paths can reach the T-Box/gateway and the CAN/CAN-FD broadcast domain. The model permits interception, modification, injection, replay, and dropping of protocol or protected-data messages. Invasive hardware, side-channel, and continuous physical-layer jamming remain outside the stated scope.

The model allows an attacker to compromise the software environment of an ordinary vehicle node and control its outgoing messages. The KGC and its master secret remain trusted, and identity-based private keys in protected storage are assumed nonextractable unless that storage boundary is breached. The attacker knows the architecture, protocol, and algorithms and controls the network but is assumed unable to break the underlying cryptographic primitives. Invasive hardware attacks, side channels, and sustained physical-layer jamming are outside the scope.

Based on the above attacker capabilities, the proposed in-vehicle secure communication scheme should satisfy the following security requirements:

Confidentiality: Because CAN and CAN FD broadcast frames, any node connected to the in-vehicle network may receive the transmitted frames. Therefore, application data, diagnostic commands, vehicle status information, and other sensitive messages should be encrypted so that only the intended communication entities can recover the plaintext.

Authentication: The T-Box and target ECU mutually authenticate before establishing an identity-bound secure session. The gateway forwards their handshake messages but is not a third authenticated endpoint in the evaluated key agreement. The session root key is bound to the identities of the two endpoints, preventing impersonation or identity substitution during negotiation.

Message Integrity: The receiver should be able to verify that a received message was generated by an authenticated communication peer and has not been modified during transmission. Any unauthorized modification to the message header, ciphertext, session identifier, sequence number, or authentication tag should be detected, and the corresponding message should be rejected.

Message Freshness: An attacker may retransmit previously captured session-negotiation messages or protected data packets to impersonate a legitimate node or trigger repeated vehicle operations. Therefore, session random values, session identifiers, sequence numbers, or other freshness information should be incorporated to ensure that previously accepted messages cannot be reused in the current session.

Session Key Security and Key Separation: The established session key should be known only to the authenticated communication parties. An attacker should not be able to derive the session key from public parameters, intercepted protocol messages, or the keys of unrelated nodes. In addition, different ECUs, sessions, communication directions, and cryptographic purposes should use independent keys so that the disclosure of one session key does not compromise other secure sessions.

### 3.3. Framework Overview

**Figure 2:**
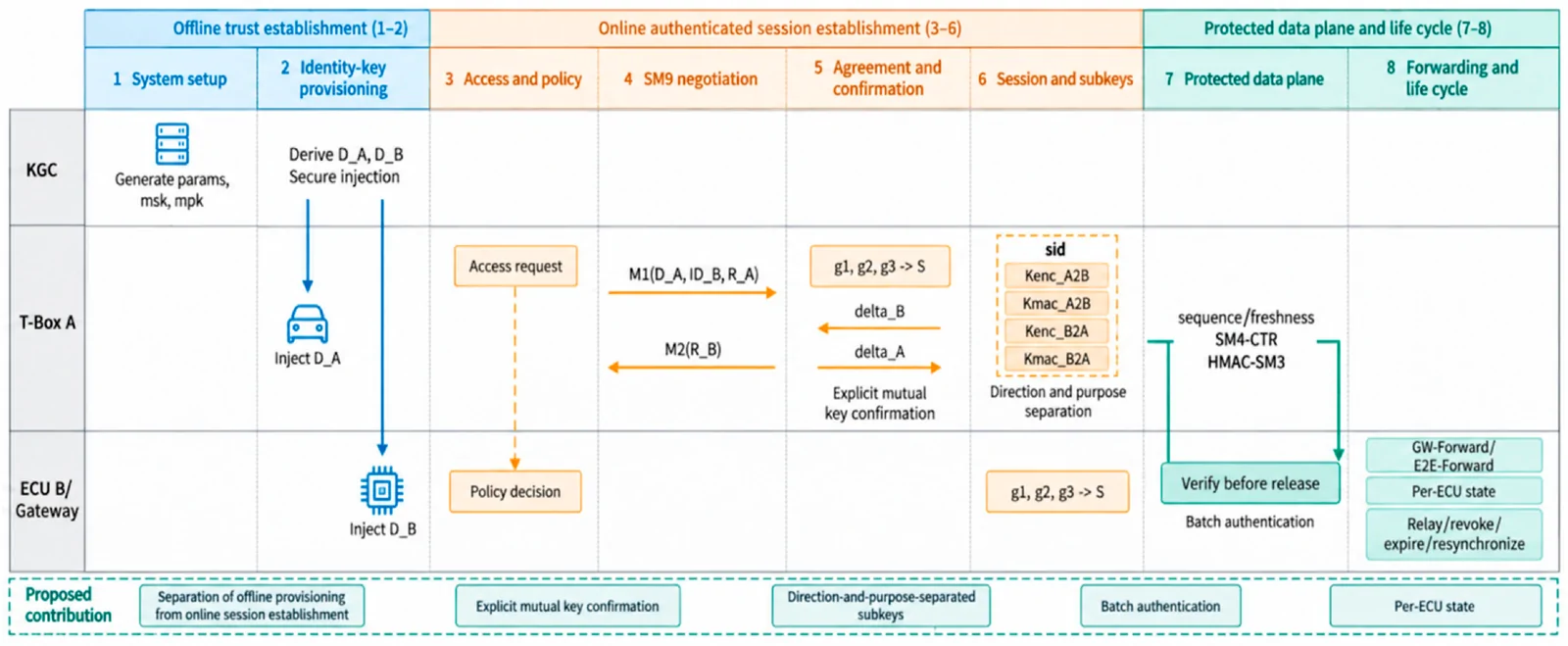
Three-lane workflow linking offline trust establishment, online authenticated session establishment, and protected data-plane/life-cycle management. Blue arrows indicate offline identity-key provisioning; orange arrows indicate access-policy and handshake/key-confirmation flows, with the dashed orange arrow showing the access-policy decision flow; the green arrow indicates delivery of protected data to receiver verification before release. Message contents are shown beside the corresponding arrows.

To satisfy the secure communication requirements between the T-Box and in-vehicle ECUs, this paper proposes a lightweight identity-based secure communication framework. Figure 2 organizes the workflow into three connected lanes and eight stages: offline trust establishment, online authenticated session establishment, and protected data-plane and life-cycle management. The offline lane covers system setup and identity-key provisioning; the online lane covers access and policy decisions, SM9 negotiation, agreement and confirmation, and session/subkey derivation; and the final lane covers protected data, forwarding, per-ECU state, and life-cycle operations. The three-lane structure separates identity-based session setup from recurring symmetric data protection while preserving the stated access-control and forwarding boundaries.

At the protocol level, the workflow links KGC initialization and identity-key provisioning with policy-controlled access, SM9 negotiation, explicit bidirectional confirmation, session-identifier and directional subkey derivation, SM4-CTR/HMAC-SM3 protection, verify-before-release processing, forwarding-mode selection, and per-ECU rekey, revocation, expiry, and resynchronization operations.

The scheme consists of the following parts:

(1) Offline Identity-Based Key Provisioning

Offline provisioning establishes the framework’s trust base. A trusted Key Generation Center (KGC) creates system parameters and long-term private keys bound to each legitimate node’s identity. The keys are securely provisioned to the T-Box, gateway, and ECUs during manufacturing or authorized maintenance. The T-Box and target ECU use their provisioned private keys for the authenticated session establishment analyzed here.

(2) Identity-Based Authenticated Session Establishment

During the online stage, the T-Box and target ECU use their long-term identity-bound private keys and fresh session randomness to derive a shared session root key and perform explicit key confirmation. Identity-based operations are confined to this stage; established-session messages use the derived symmetric subkeys.

(3) Protected Data-Plane and Life-cycle Management

In the third phase, an established session supports protected data exchange. Independent direction- and purpose-specific subkeys protect service messages, while the session identifier and freshness state bind accepted messages to the current session. Established-session traffic uses symmetric encryption and authentication rather than repeating identity-based operations for each message.

The third phase also covers T-Box multi-session management and secure forwarding. The T-Box maintains an independent security context for each target ECU and may reuse a session only while its lifetime, revocation version, and local policy remain valid. Depending on the deployment boundary, the framework supports T-Box decrypt-and-forward via the gateway and end-to-end ciphertext-forwarding modes for communication between off-board services and in-vehicle ECUs.

Section 3.4 and Section 3.5 detail the first two phases. Section 3.6 and Section 3.7 jointly describe the third phase, covering data-plane protection, secure forwarding, and session life-cycle management. The protocol is instantiated using an SM9-based identity-bound construction, while Table 2 consolidates the notation and parameter definitions used throughout the scheme.

### 3.4. Offline Identity-Based Key Provisioning

(1) System initialization. In the system initialization stage, the trusted Key Generation Center (KGC) generates the system master private key *msk* and master public key *mpk* and publishes the system parameters *params*. These parameters include bilinear-pairing group parameters, generators P_1_ and P_2_, hash-to-group mapping functions, and related values. The system parameters *params* and master public key *mpk* serve as the public trust foundation of the in-vehicle security domain. They can be preconfigured in relevant nodes during vehicle production or distributed through a software update link with integrity and authenticity protection.

This process supports subsequent private-key generation, identity binding, and session-key agreement without the certificate issuance, distribution, and verification required by conventional public-key infrastructures.

(2) Private-key generation. For the unique identity *ID_i_* of each vehicular node, the system generates the corresponding SM9 private key based on its identity information. To prevent the same identity from reusing the same mapping input under different cryptographic functions, a public function tag *tag* is introduced in the identity hashing stage to distinguish the functional domains corresponding to different purposes. Let the system master private key be $s\in Z_{n}^{*}$, the base point in the system parameters be *P*_1_, and the group order be *n*. The node private-key generation process is as follows:

First, calculate the hash mapping value according to the node identity and function tag:

h = *H*_1_(*ID*‖*tag*,*n*) (1)

where $H_{1}:\{{0,1\}}^{*}\to Z_{n}^{*}$ denotes the hash mapping function, whose output is a nonzero integer modulo *n*; the public usage *tag* can be represented by a fixed hexadecimal constant. For example, one can define

*tag_KA_* = 0x01,*tag_SIG_* = 0x02,*tag_ENC_* = 0x03 (2)

corresponding to key agreement, digital signature, and encryption functions, respectively. In this way, the same node identity obtains mutually independent identity mapping inputs under different security functions. Then, the intermediate value is calculated:

*t*_1_ = (*s* + *h*)^−1^(mod *n*) (3)

(*s* + *h*) ⋅ *t*_1_ = 1 mod *n* (4)

where (*s* + *h*)^−1^ mod *n* denotes the multiplicative inverse of the integer *s* + *h* modulo *n*; that is, there exists (*s* + *h*) ⋅ *t*_1_≡1(mod *n*).

After obtaining *t*_1_, further calculate the scalar.

*k*_i_ = *s* ⋅ *t*_1_ mod *n* (5)

Finally, the node private key is

*D*_i_ = *k*_i_*P*_1_ = (*s* ⋅ *t*_1_)*P*_1_ (6)

where *k*_i_*P*_1_ denotes scalar multiplication on the elliptic-curve group, namely multiplying the base point *P*_1_ by an integer scalar to obtain a point *D*_i_ on the curve. Therefore, *D_i_* is essentially not an ordinary integer but a point on the elliptic-curve group G_1_, which can be written as

(7) $$D_{i}=(x_{D_{i}},y_{D_{i}})$$

where $x_{D_{i}}$ and $y_{D_{i}}$ denote the coordinate representation of the curve point. This point constitutes the private key of the ECU node under the specified function tag *tag*.

The ECU node private-key generation algorithm is shown in Algorithm 1. It first calculates the hash value *h* according to the identity information and function tag, then combines it with the system master private key *s* to calculate the modular inverse *t*_1_, generates the scalar *k*_i_, and finally obtains the private key *D*_i_ through elliptic-curve scalar multiplication. Because different tags *tag* lead to different *h* values and, consequently, different derived values *t*_1_, *k*_i_ and *D*_i_, private keys generated under different security functions remain independent even for the same node identity *ID*_i_. This realizes functional-domain separation and avoids cross-purpose parameter reuse.
**Algorithm 1:** SM9 identity-private-key extraction for vehicle node iInput: *ID*_i_, public function label *tag*_ID_, master private scalar s, system parameters (*P*_1_,*n*,H_1_) Output: Long-term identity private point *D*_i_ Precondition: The KGC is trusted; s never leaves the KGC security boundary. 1.Compute $h\;=\;H_{1}({\mathrm{ID}}_{i}||{\mathrm{tag}}_{\mathrm{ID}},n);\;\mathrm{abort}\;\mathrm{if}\;h\;\mathrm{not}\;\mathrm{in}\;Z_{n}^{*}$. 2.Compute *t*_1_ = (s + h)^−1^mod *n*; abort if the inverse does not exist. 3.Compute *k*_i_ = *s* ⋅ *t*_1_ mod *n*. 4.Compute *D*_i_ = k_i_P_1_ in G_1_ 5.Provision *D*_i_ and the associated identity/function metadata through the protected manufacturing or maintenance channel.
Here, the operation [*k_i_*]*P*_1_ in step 4 denotes scalar multiplication on the elliptic-curve group *G*_1_, multiplying the base point *P*_1_ by an integer *k*_i_ and outputting a curve point *D*_i_.


During deployment, the KGC generates identity-bound private keys from the system master key and node identifiers and provisions them to the relevant nodes. Private-key use and storage should be confined to protected environments to limit exposure in ordinary software. Distribution, renewal, revocation, and auditing must then be managed throughout each key’s life cycle.

### 3.5. Identity-Based Authenticated Session Establishment

The online stage performs mutual identity authentication and session-key agreement after offline provisioning of the long-term node keys. The T-Box and target ECU exchange fresh ephemeral public values, derive the same SM9-based shared term, bind the ordered identities and transcript in KDF-SM3, and confirm the result with direction-specific authentication tags. The identities and fresh session values are therefore included in each session’s key-derivation context. The resulting session supports the remote-diagnostic, parameter-configuration, protected-control, and OTA flows considered in this work. The procedure is detailed below:

Let A denote the T-Box and B the target ECU. Online establishment comprises four steps: session initialization, SM9 negotiation-parameter generation, shared session-key computation, and bidirectional key confirmation.

During session initialization, T-Box A sends a negotiation request message M_1_ to node B:

M_1_ = KA_Req_(*ID*_A_,*ID*_B_,*R*_A_) (8)

where *ID*_A_ and *ID*_B_ denote the identities of the two communicating parties, and the negotiation parameter *R*_A_ is generated by node A for the current session. After receiving the request, B sends the following response message M_2_ to A:

M_2_ = KA_Resp_(*R*_B_) (9)

where *R*_B_ denotes the negotiation parameter generated by node B. These messages initialize the session for subsequent shared-term construction and key derivation. During SM9 negotiation-parameter generation, each party selects a fresh random scalar for the current session. Let the system master private key be $s\in Z_{n}^{*}$ and the system master public key be

*P_pub_* = *sP*_1_ (10)

where *P*_1_ and *P*_2_ are base points on the corresponding groups, and the mapping *e*:*G*_1_ × *G*_2_ → *G_T_* denotes the bilinear pairing. For any node U ∈ {A,B} with an identity identifier, the identity hash value *ID*_U_ is defined as

H_U_ = H_1_(*ID*_U_‖*tag*,*n*) (11)

The public identity point on the elliptic curve is further constructed.

*Q*_U_ = h_U_*P*_1_ + *P_pub_* = (h_U_ + *s*)*P*_1_ (12)

On this basis, node A selects a random number $r_{A}\in Z_{n}^{*}$ and calculates the negotiation parameter:

*R*_A_ = *r*_A_*Q*_B_ (13)

Node B selects a random number $r_{B}\in Z_{n}^{*}$ and calculates the negotiation parameter:

*R*_B_ = *r*_B_*Q*_A_ (14)

Because the generation of *R*_A_ and *R*_B_ depends on both local random numbers and identities, the resulting negotiation parameters are session-specific and bound to the participants, supporting separation across sessions and node pairs.

Next, in the vehicular negotiation-parameter generation stage, the two parties combine their own private keys and local random numbers to construct shared intermediate values based on the bilinear pairing. After receiving *R*_B_, node A calculates

(15) $$g_{1}^{A}=e\left(R_{B},D_{A}\right)=g^{r_{B}}$$

(16) $$g_{2}^{A}=e(P_{\mathrm{pub}},P_{2}{)}^{r_{A}}=g^{r_{A}}$$

(17) $$g_{3}^{A}=(g_{1}^{A}{)}^{r_{A}}=g^{r_{A}r_{B}}$$

Node B correspondingly calculates:

(18) $$g_{1}^{B}={e(P_{\mathrm{pub}},P_{2})}^{r_{B}}=g^{r_{B}}$$

(19) $$g_{2}^{B}=e(R_{A},D_{B})={\;g}^{r_{A}}$$

(20) $$g_{3}^{B}=(g_{2}^{B}{)}^{r_{B}}=g^{r_{A}r_{B}}$$

where *D*_A_ and *D*_B_ denote the private keys generated and securely injected for nodes A and B during the offline stage. Thus, the online stage does not regenerate node private keys but constructs the shared intermediate value by combining the identity private keys established offline with the one-time random values of the current session.

After constructing the shared intermediate values, both parties use an SM3-based key derivation function to generate their local session root keys.

Node A calculates:

(21) $$S_{A}=\mathrm{KD}F_{\mathrm{SM}3}\left(ID_{A}\|ID_{B}\|R_{A}\|R_{B}\|g_{1}^{A}\|g_{2}^{A}\|g_{3}^{A},k_{s}\right)$$

Node B calculates:

(22) $$S_{B}=\mathrm{KD}F_{\mathrm{SM}3}\left(ID_{A}\|ID_{B}\|R_{A}\|R_{B}\|g_{1}^{B}\|g_{2}^{B}\|g_{3}^{B},k_{s}\right)$$

where *k_s_* denotes the output length of the session root key. Under correct protocol execution, the following should hold:

*S*_A_ = *S*_B_ = *S* (23)

The resulting *S* is the session root key shared by the T-Box and the target ECU in the current session.

Finally, the parties use authentication tags derived from the session root key *S* to confirm that they obtained the same result. Let the confirmation key derived from *S* be

*K_c_* = *KDF_SM_*_3_(*S*‖“confirm”,*k*_c_) (24)

where *k*_c_ denotes the confirmation-key length, and the string “*confirm*” is used as a usage identifier to realize domain separation between the key-confirmation process and the subsequent service data protection process.

On this basis, node B uses its local session root key *S_B_* to derive the confirmation key and then calculates the authentication tag.

(25) $$\delta_{B}={\mathrm{HMAC-SM}3}_{K_{c}}^{B}\left(ID_{A}\|ID_{B}\|R_{A}\|R_{B}\|1\right)$$

Node B sends this tag to A. After receiving it, A uses its local session root key *S*_A_ to derive the confirmation key $K_{c}^{A}$ and recalculates

(26) $$\delta_{B}^{\prime }={\mathrm{HMAC-SM}3}_{K_{c}}^{A}\left(ID_{A}\left|\left|ID_{B}\right|\right|R_{A}\left|\left|R_{B}\right|\right|1\right)$$

If the condition $\delta_{B}^{\prime }=\delta_{B}$ is satisfied, node A passes verification, indicating that node B has correctly obtained a shared session key consistent with node A.

Subsequently, node A uses the same method and the confirmation key $K_{c}^{A}$ to calculate a return authentication tag *δ*_A_ and sends it to node B. After receiving

(27) $$\delta_{A}={\mathrm{HMAC-SM}3}_{K_{c}}^{A}\left(ID_{A}\|ID_{B}\|R_{A}\|R_{B}\|0\right)$$

node B recalculates the tag:

(28) $$\delta_{A}^{\prime }={\mathrm{HMAC-SM}3}_{\mathrm{Kc}}^{B}\left(ID_{A}\|ID_{B}\|R_{A}\|R_{B}\|0\right)$$

using its local confirmation key $K_{c}^{B}$. If the condition $\delta_{A}^{\prime }=\delta_{A}$ is satisfied, node A has also correctly obtained the same shared session key as node B. At this point, both parties complete bidirectional key confirmation, and the secure session is established; otherwise, node B determines that session establishment has failed and terminates the current session.

The constants 1 and 0 identify the confirmation messages initiated by B and A, respectively. Including distinct direction labels prevents a valid confirmation tag from being reused in the opposite direction under the same transcript. Together with fresh ephemeral values and transcript binding, this construction provides explicit bidirectional key confirmation within the stated model.

Algorithm 2 summarizes SM9-based online session establishment between the T-Box and target ECU.
**Algorithm 2:** Authenticated SM9 session establishment between T-Box A and ECU BInput: system public parameters *params*, T-Box identity *ID*_A_, ECU identity *ID*_B_, T-Box private key *d*_A_, ECU private key *d*_B_ Output: session root key *S*, confirmation key *K_c_*
 1:The T-Box selects a random number r_A_ and calculates a temporary public value R_A_. 2:The T-Box sends (*ID*_A_,*R*_A_) to the ECU. 3:The ECU selects a random number r_B_ and calculates a temporary public value *R*_B_. 4:The ECU calculates the shared intermediate value according to (*ID*_A_,*R*_A_,*ID*_B_,*R*_B_,*d*_B_,*r*_B_) and derives the session root key *S*_B_. 5:The ECU derives the confirmation key $K_{c}^{B}$ according to *S*_B_ and generates an authentication tag *δ*_B_. 6:The ECU sends the message (*ID*_B_,*R*_B_,*δ*_B_) to the T-Box. 7:The T-Box calculates the shared intermediate value according to (*ID*_A_,*R*_A_,*ID*_B_,*R*_B_,*d*_A_,*r*_A_) and derives the session root key *S*_A_. 8:The T-Box derives the confirmation key $K_{c}^{A}$ according to *S*_A_ and verifies the tag *δ*_B_. 9:If verification succeeds, the T-Box generates an authentication tag *δ*_A_ and sends it to the ECU. 10:The ECU uses $K_{c}^{B}$ to verify *δ*_A_. 11:If verification succeeds, set *S* ← *S*_A_ = *S*_B_, $K_{c}\leftarrow K_{c}^{A}=K_{c}^{B}$. 12:Return *S*, *K_c_*.


### 3.6. Lightweight Data-Plane Protection

Once the T-Box and target ECU have established a session root key *S*, service traffic enters the data plane. This stage carries diagnostic commands, control messages, configuration data, status responses, and OTA data under the established session. The handshake authenticates the participants and establishes *S*; the data plane uses subkeys derived from *S* to protect recurring messages. Separate encryption and authentication subkeys support confidentiality, integrity, source authentication, and replay protection without reusing the root key directly. In this way, the identity binding established during the handshake extends to protected service traffic.

The mechanism has two parts. It first derives a session identifier and purpose-specific data-plane keys from current session information. It then encrypts and authenticates service messages in a common PDU format.

#### 3.6.1. Subkey Derivation for Vehicular Bidirectional Data Communication

To establish a clear correspondence between subsequent data-plane communication and the current established session, the session identifier *sid* is defined as

*sid* = *SM*3(*SM*3(*ID*_A_)‖*SM*3(*ID*_B_)‖*Encode*(*R_A_*)‖*Encode*(*R_B_*)) (29)

where ID_A_ and ID_B_ denote the identity identifiers of the T-Box and target ECU, respectively; *R*_A_ and *R*_B_ denote the negotiation parameters exchanged by both parties during online session establishment; and the deterministic encoding function *Encode*(∙) converts the negotiation parameters into a unique byte-string representation according to unified rules, ensuring that both communicating parties obtain consistent hash, key-derivation, and authentication results for the same inputs. Because the value *sid* is jointly determined by the identities of both parties and the current session parameters, it corresponds one-to-one with this session. Different communicating parties or different negotiation parameters derive different identifiers *sid*. In subsequent communication, *sid* serves both as the session index field of data-plane messages and as part of the subkey derivation input to strengthen the binding between the key and the current session.

On this basis, this paper uses an SM3-based key derivation function to expand the session root key *S*. Let *label_i_* denote the usage identifier of the i-th subkey and *n_i_* denote its output length; the corresponding subkey is defined as

*K_i_* = *KDF_SM_*_3_(*S*‖*sid*‖*label_i_*‖*n_i_*) (30)

By introducing the session identifier *sid* and usage label *label_i_* into the derivation function, subkeys derived from the same session root key are separated by purpose, avoiding direct reuse of the same root key for different functions. For vehicular bidirectional data communication, this paper derives the following four subkeys:

(31) $$K_{\mathrm{enc}}^{A\to B}=\mathrm{KD}F_{\mathrm{SM}3}(S||\mathrm{sid}||“\mathrm{ENC}\_A2B”||16)$$

(32) $$K_{\mathrm{mac}}^{A\to B}=\mathrm{KD}F_{\mathrm{SM}3}(S||\mathrm{sid}||“\mathrm{MAC}\_A2B”||32)$$

(33) $$K_{\mathrm{enc}}^{B\to A}=\mathrm{KD}F_{\mathrm{SM}3}(S||\mathrm{sid}||“\mathrm{ENC}\_B2A”||16)$$

(34) $$K_{\mathrm{mac}}^{B\to A}=\mathrm{KD}F_{\mathrm{SM}3}(S||\mathrm{sid}||“\mathrm{MAC}\_B2A”||32)$$

Here, the corresponding value denotes the A-to-B data encryption key $K_{\mathrm{enc}}^{A\to B}$ derived from the session root key *S*. Its derivation input includes the current session identifier *sid* and functional direction identifier, ensuring that the key is used only for A-to-B data encryption in the current session. The value 16 in the formula indicates that the derived key output length is 16 bytes, corresponding to the 128-bit key length required by SM4. Similarly, $K_{\mathrm{mac}}^{A\to B}$, $K_{\mathrm{enc}}^{B\to A}$, and $K_{\mathrm{mac}}^{B\to A}$ denote the A-to-B data authentication key, the B-to-A data encryption key, and the B-to-A data authentication key in the current session, thereby separating subkeys across different communication directions and security uses. Based on the above session identifier generation process and subkey derivation relationship, the session binding and subkey expansion process for vehicular bidirectional data communication is summarized in Algorithm 3.
**Algorithm 3:** Session identifier and directional subkey derivationInput: session root key *S*, identity identifiers ID_A_, ID_B_, negotiation parameters *R*_A_, *R*_B_Output: session identifier *sid* and subkeys $K_{\mathrm{enc}}^{A\to B},K_{\mathrm{mac}}^{A\to B},K_{\mathrm{enc}}^{B\to A},K_{\mathrm{mac}}^{B\to A}$
Precondition: The KDF input encoding and public labels are identical at both endpoints.
 1:Encode R_A_ and R_B_ using the unique protocol encoding. 2:Compute *sid* = SM3(SM3(*ID*_A_)‖SM3(*ID*_B_)‖*Encode*(*R*_A_)‖*Encode*(*R*_B_)). 3:Derive $K_{\mathrm{enc}}^{A\to B}$ using label ENC_A→B_ and output length 16 bytes. 4:Derive $K_{\mathrm{mac}}^{A\to B}$ using label MAC_A→B_ and output length 32 bytes. 5:Derive $K_{\mathrm{enc}}^{B\to A}$ using label ENC_B→A_ and output length 16 bytes. 6:Derive $K_{\mathrm{mac}}^{B\to A}$ using label MAC_B→A_ and output length 32 bytes. 7:Create an isolated ST[*ID*_B_] entry and return sid and the four subkeys.

#### 3.6.2. Vehicular Data Encryption Mechanism

After subkey derivation, data-plane communication uses an encrypt-then-authenticate structure to provide confidentiality, integrity, source authentication, and replay protection for service and control data. A common protocol data unit (PDU) format carries control, service, and keepalive messages. Its core fields include message type *type*, session identifier *sid*, sequence number *seq*, direction bit *dir*, plaintext length field *len*, initialization vector or random number *nonce*, ciphertext *ciphertex*, and authentication tag *τ*.

The sender first constructs the message header:

*header* = (*type*‖*sid*‖*seq*‖*dir*‖*len*) (35)

Let the encryption key be $K_{\mathrm{enc}}^{\mathrm{dir}}$ and authentication key be $K_{\mathrm{mac}}^{\mathrm{dir}}$ in the current direction; the sender then calculates

(36) $$\mathrm{ciphertext}=\mathrm{SM}4\mathrm{-CTR}\left(K_{\mathrm{enc}}^{\mathrm{dir}},\mathrm{nonce},\mathrm{plaintext}\right)$$

(37) $${\mathrm{tag}=\mathrm{HMAC-SM}3}_{K_{\mathrm{mac}}^{\mathrm{dir}}}\left(\mathrm{header}\;||\;\mathrm{nonce}\;||\;\mathrm{ciphertext}\right)$$

The final transmitted data-plane message can be expressed as:

*PDU* = *header*‖*nonce*‖*ciphertext*‖*τ* (38)

This paper adopts SM4-CTR as the data-plane encryption algorithm and HMAC-SM3 as the authentication algorithm. SM4-CTR accommodates segmented transmission and arbitrary-length payloads in CAN/CAN FD scenarios. HMAC-SM3 is used to jointly authenticate the header, random number, and ciphertext, allowing the receiver to detect tampering and authenticate the sender, as shown in Algorithm 4.
**Algorithm 4:** Encrypt-then-authenticate sender processingInput: *sid*, *type*, *seq*, *dir*, *plaintext*, $K_{\mathrm{enc}}^{\mathrm{dir}}$, $K_{\mathrm{mac}}^{\mathrm{dir}}$, unique *nonce* Output: Authenticated data-plane PDU Precondition: sid is valid, directional keys are active, and nonce uniqueness is guaranteed.
 1:Atomically reserve the next sequence number for the current direction; abort if rekeying is required. 2:Set *len* ← |*plaintext*| and *header*←(*type* ‖ *sid* ‖ *seq* ‖ *dir* ‖ *len*). 3:Compute $\mathrm{ciphertext}\leftarrow \mathrm{SM}4\mathrm{-CTR}\left(K_{\mathrm{enc}}^{\mathrm{dir}},\;\mathrm{nonce},\;\mathrm{plaintext}\right)$. 4:Compute $\tau \leftarrow {\mathrm{HMAC}\mathrm{-SM}3}_{K_{\mathrm{mac}}^{\mathrm{dir}}}\left(\mathrm{header}||\mathrm{nonce}||\mathrm{ciphertext}\right)$, truncated only to the negotiated profile length. 5:Construct *PDU*←*header* ‖ *nonce* ‖ *ciphertext* ‖ *τ*. 6:Commit the sending state and transmit PDU; never reuse nonce under the same $K_{\mathrm{enc}}^{\mathrm{dir}}.$

After receiving a data-plane message, the receiver verifies it before decryption. It first checks whether *sid* exists and whether the corresponding session is still valid. It then verifies whether *seq* satisfies the current policy according to the locally maintained freshness state. After the session and freshness checks pass, it uses the authentication key in the corresponding direction to recalculate the expected authentication tag.

(39) $$\tau^{\prime }={\mathrm{HMAC-SM}3}_{K_{\mathrm{mac}}^{\mathrm{dir}}}\left(\mathrm{header}\|\mathrm{nonce}\|\mathrm{ciphertext}\right)$$

It then compares the result with the received tag *tag*. If *tag*′ = *tag* is satisfied, the message has not been tampered with during transmission and comes from a legitimate sender holding the correct authentication key. The receiver then decrypts it using the encryption key in the corresponding direction.

(40) $$\mathrm{plaintext}=\mathrm{Dec}\left(K_{\mathrm{enc}}^{\mathrm{dir}},\mathrm{nonce},\mathrm{ciphertext}\right)$$

The recovered plaintext is then delivered to the upper-layer application for processing, as shown in Algorithm 5.
**Algorithm 5:** Verify-before-release receiver processingInput: Received PDU, ST, expected direction, negotiated authentication-tag length Output: Plaintext or reject Precondition: No plaintext is released before authentication and freshness checks succeed.
 1:Parse *header*, *nonce*, *ciphertext*, and *τ*; reject malformed lengths or encodings. 2:Look up *ST*[*ID*_B_] by *sid*; reject unknown, expired, revoked, or wrong-direction sessions. 3:Perform an atomic replay-window check for seq without committing state; reject duplicates or out-of-window values. 4:Compute $\tau \prime \;=\;{\mathrm{HMAC-SM}3}_{K_{\mathrm{mac}}^{\mathrm{dir}}}\left(\mathrm{header}\;||\;\mathrm{nonce}\;||\;\mathrm{ciphertext}\right)$. 5:If ConstantTimeEqual(*τ*′, *τ*) is false, reject without releasing plaintext. 6:Decrypt $\mathrm{plaintext}=\mathrm{SM}4\mathrm{-CTR-Dec}\left(K_{\mathrm{enc}}^{\mathrm{dir}},\mathrm{nonce},\mathrm{ciphertext}\right)$. 7:Verify len and application framing; reject inconsistencies. 8:Atomically commit the freshness update, then deliver plaintext to the upper layer.

After session establishment and key confirmation, the data-plane mechanism uses the session root key S. It expands S into direction- and purpose-specific subkeys using the session identifier sid and usage labels. The sender then encrypts the payload using SM4-CTR and jointly authenticates the header, random number, and ciphertext using HMAC-SM3. The receiver completes message verification and plaintext recovery only after verification.

#### 3.6.3. Freshness, Replay, and Rekey Policy

For the standard PDU, seq is a 32-bit unsigned integer. Each direction initializes its transmit sequence to 1; value 0 is never transmitted, and the receiver initializes its highest authenticated value H and 64-bit replay bitmap to zero. A packet is structurally checked and bound to the expected sid and direction, its HMAC-SM3 tag is verified in constant time, and its plaintext is kept internal until the replay state is committed. If *seq* > H and seq−H ≤ 4096, the bitmap is shifted and seq becomes the new H. If *seq* ≤ H, the packet is accepted only when H−seq < 64 and the corresponding bit has not already been set. A duplicate or a value outside the 64-entry window is rejected.

Cryptographic verification precedes freshness mutation, while the final check-and-mark operation is executed under the per-session lock. Thus, concurrent deliveries of the same authenticated packet have exactly one successful commit and plaintext release. An authenticated forward jump greater than 4096 does not move the window; it requires authenticated resynchronization or a fresh session. The compact classical-CAN profile is treated separately because its sequence is implicit rather than carried in each frame: it is strictly ordered within a batch, releases no plaintext before batch authentication, and uses the authenticated three-frame control message after loss or desynchronization.

Wraparound is prohibited. Rekeying is mandatory at *T_rekey_* = 2^32^−4096 = 4,294,963,200, so the final 4096 counter values are reserved and never used under the old directional keys. Expiry, reaching *T_rekey_*, persistent desynchronization, an authenticated rekey instruction, endpoint reboot, ECU replacement, or revocation invalidates the old session and requires a complete SM9 exchange and bidirectional key confirmation, which generates a new sid and new directional subkeys. After reboot, volatile session keys, sid, counters, the replay bitmap, and the resynchronization counter are discarded; the sequence is never reset to 1 under the old key.

### 3.7. T-Box Multi-Session Management and Secure Forwarding

This section describes secure forwarding and session life-cycle management. For off-board requests, the T-Box parses the target ECU and service type, applies local access-control policy, and locates the corresponding session. It reuses a valid session when its lifetime, revocation state, and policy permit; otherwise, it initiates session establishment and derives new subkeys. The T-Box then selects a forwarding mode, forwards the protected message, and updates the session state. In deployment, these functions require request parsing, target location, access control, session maintenance, and message forwarding.

To support concurrent access to multiple ECUs, the T-Box uses the target ECU identifier *ID_B_* as an index and maintains the corresponding session context in the session state table ST, namely

(41) $$\mathrm{ST}[{ID}_{B}]=(\mathrm{sid},K_{\mathrm{enc}}^{A\to B},K_{\mathrm{mac}}^{A\to B},K_{\mathrm{enc}}^{B\to A},K_{\mathrm{mac}}^{B\to A},\mathrm{seq},\mathrm{fresh},\mathrm{lifetime})$$

where *sid* denotes the session identifier; $K_{\mathrm{enc}}^{A\to B}$, $K_{\mathrm{mac}}^{A\to B}$, $K_{\mathrm{enc}}^{B\to A}$, and $K_{\mathrm{mac}}^{B\to A}$ denote bidirectional encryption and authentication subkeys; seq denotes the sequence-number state; fresh denotes the freshness state; and lifetime denotes the session validity period. This state-maintenance method enables the T-Box to quickly locate the session parameters corresponding to a target ECU and complete session reuse, state update, expiration judgment, and multi-session isolation.

Considering service requirements and system trust boundaries, this paper specifies two forwarding modes. In GW-Forward (T-Box decrypt-and-forward via the gateway), the T-Box verifies and decapsulates an incoming service message, re-encapsulates it under the target ECU’s session context, and sends the protected message through the in-vehicle gateway. The gateway routes the message but is not a party to the T-Box–ECU key agreement. In E2E-Forward, the T-Box and gateway route handshake and ciphertext messages without decrypting the protected service payload. The former supports access control, auditing, and anomaly detection at the T-Box, while the latter reduces service-plaintext exposure at that point. The processing flow comprises request parsing, access-control checking, session retrieval or establishment, forwarding-mode selection, protected forwarding, and session-state update.

#### Operational Rekey, Revocation, and ECU Replacement

Life-cycle changes are carried in an OEM/KGC-authorized control record that binds a strictly increasing version, action, ECU identity, optional replacement identity, and reason using canonical encoding and domain separation. The executable reference implementation authenticates this record with HMAC-SM3 under a separately provisioned management key; a production deployment may instead verify an OEM/KGC digital signature inside an HSM. In either case, an invalid tag or a non-increasing version is rejected, and the verified record is applied atomically.

After a T-Box or gateway successfully receives and verifies a revocation, every active session for that ECU identity is immediately marked revoked, further traffic on retained references is rejected, and new handshakes for that identity are blocked. This is a local post-verification guarantee; an offline vehicle may still experience propagation delay. A rekey instruction invalidates the active context but does not revoke the identity, so the next authorized access performs a complete SM9 handshake and explicit confirmation.

For ECU replacement, the old identity is revoked, and the replacement is provisioned offline by the KGC with a new identity-bound private key. It cannot reuse the old sid, session keys, sequence state, or replay bitmap and becomes operational only after a new confirmed session is established. The Python reference invalidates and drops old key references but cannot guarantee physical zeroization of immutable byte objects; production secure erasure therefore remains an HSM or secure-storage requirement.

## 4. Security Verification

### 4.1. ProVerif Tool and Symbolic Model

ProVerif is an automated verifier for cryptographic protocols expressed in the applied pi calculus [33]. It analyzes secrecy and correspondence properties under the Dolev–Yao attacker model by translating protocol processes into Horn clauses. The verification workflow comprises three steps: protocol and query specification, automatic symbolic analysis over unbounded concurrent sessions, and interpretation of the resulting true, false, or unproved judgments. In this work, ProVerif 2.05 is used to verify the session-establishment and data-plane security properties of the proposed identity-based secure communication scheme.

The public UDP/CAN communication path is modeled as an attacker-controlled channel. The adversary can intercept, modify, delay, replay, delete, and inject arbitrary protocol messages and can compose any term derivable from its current knowledge. The KGC master secret and the long-term private keys of the honest T-Box A and target ECU B remain confidential. To evaluate multi-session isolation against an unrelated-node compromise, the private key of a third ECU C is explicitly disclosed to the attacker. The cryptographic hash function, key-derivation function, message-authentication mechanism, and symmetric encryption algorithm are modeled as perfect cryptographic primitives. The identity-based authenticated key-agreement operation is abstracted using identity-key extraction, identity-bound ephemeral public values, and an agreement equation stating that A and B derive the same session root key only when their identities, long-term credentials, and fresh ephemeral values are correctly matched.

The four session-establishment messages, M_1_, M_2_, KC1, and KC2, are modeled explicitly. The key-confirmation tags bind *ID*_A_, *ID*_B_, *R*_A_, *R*_B_, and distinct direction labels to the confirmation key. After successful bidirectional confirmation, the model derives the session identifier sid and four direction- and purpose-specific subkeys, namely Kenc-A, Kmac-A, Kenc-B, and Kmac-B. A representative protected PDU in each direction contains the message type, session identifier, sequence number, direction indicator, payload length, nonce, ciphertext, and message-authentication tag. The tag covers the complete header, nonce, and ciphertext. Before delivering the recovered plaintext, the receiver verifies the authentication tag and performs a serialized check-and-insert operation on the (sid, seq, dir) replay state.

### 4.2. Security-Property Verification

Table 3 summarizes the outcomes of the eleven ProVerif queries. Under the declared Dolev–Yao attacker model and perfect-cryptography abstraction, all queries return true for the modeled secrecy and injective-correspondence properties. These results are limited to the symbolic model and do not constitute a computational-security proof of the implemented pairing-based construction.

Authentication verification. Mutual authentication and session agreement are expressed as two injective correspondence queries. Q1 requires every T-Box acceptance event to correspond to a unique ECU begin event with the same identities, ephemeral values, and session root key. Q2 requires every ECU acceptance event to correspond to a unique T-Box begin event for the same transcript. These queries jointly detect impersonation, replayed handshakes, unknown-key-share behavior, and failure of explicit key confirmation within the symbolic model.

Session-key confidentiality and key-isolation verification. Five private probe values are protected by the session root key and four derived subkeys, respectively. Queries Q3–Q7 ask whether the attacker can recover any probe. Under the perfect-cryptography abstraction, a true non-derivability result means that the corresponding key is not derivable from public parameters, recorded protocol messages, or the compromised key of ECU C. Distinct public KDF labels and sid are included in every derivation term, thereby modeling separation by session, direction, and cryptographic purpose.

Data confidentiality verification. The private service payloads service_A→B_ and service_B→A_ are encrypted with their corresponding subkeys before being released on the public channel. Q8 and Q9 test whether the attacker can derive either plaintext from the complete network trace.

Integrity and Freshness. Q10 and Q11 use injective correspondences between PDU-receive and PDU-send events. The event arguments include the two identities, sid, seq, and recovered plaintext. Consequently, a successful result requires every accepted PDU to match one unique authentic send event; changes to the header, direction, sequence number, nonce, ciphertext, or tag cannot lead to acceptance, and the same authenticated (sid, seq, dir) tuple cannot be accepted twice in the modeled receive state.

### 4.3. Computational Security Argument and Explicit Claim Boundary

The prototype implements an SM9-based pairing variant with explicit key confirmation. The software backend uses BN254/alt_bn128-style group parameters and has not undergone official GM/T 0044 known-answer or interoperability testing. The following analysis is a computational proof sketch for the implemented construction; it is not a complete reduction or a standard-conformance claim.

Security experiment and assumptions. The adversary controls the network, starts concurrent sessions, replays or replaces handshake values, and may reveal completed session keys. The KGC and random-number generator are trusted during an uncompromised run. Every received point must be non-infinity, on-curve, and in the intended prime-order subgroup; each ephemeral scalar is sampled uniformly from [1, n−1] and erased after use. The ordered transcript (*ID*_A_, *ID*_B_, *R*_A_, *R*_B_, role) is encoded unambiguously and bound to a role-labeled confirmation tag. The sketch treats KDF-SM3 as a random-oracle/secure extractor, HMAC-SM3 as unforgeable, and CDH in G_T_ as hard.

Algebraic correctness. For *D*_U_ = *s*(*s* + *h*_U_)^−1^*P*_D_, Q_U_ = (*s* + *h*_U_)*P*_R_, and g = e(*sP*_R_,*P*_D_), bilinearity gives e(*R*_B_,*D*_A_) = e(*r*_B_(*s* + *h*_A_)*P*_R_, $s(s+h_{A}{)}^{-1}P_{D})=g^{r_{B}}$. Similarly, $e(R_{A},D_{B})=g^{r_{A}}$; both parties then obtain $g^{r_{A}r_{B}}$ and derive the same root from the same ordered transcript and term triple.

Game sequence. G_0_ is the real protocol. In *G*_1_, the challenger aborts on invalid, infinity, or non-subgroup points and on an honest ephemeral-scalar collision. In *G*_2_, for a fresh accepted transcript, the KDF output is replaced by an independent random string unless the adversary queries the exact transcript including $g^{r_{A}r_{B}}$. In *G*_3_, an adversary making that query without an allowed reveal yields the missing CDH target from ($g^{r_{A}},g^{r_{B}}$). In *G*_4_, an unmatched acceptance requires either a transcript/KDF collision or a fresh HMAC-SM3 forgery under the dedicated confirmation key. Consequently, the distinguishing and impersonation advantages are bounded qualitatively by the corresponding CDH, KDF/random-oracle, HMAC-unforgeability, encoding-collision, validation, and ephemeral-collision terms. A publication claiming a full theorem would still need to specify the exact bilinear-group assumption and provide a simulator and quantitative reduction.

KCI boundary. Disclosure of *D*_A_ trivially permits impersonation of A. It does not immediately reveal a construction for impersonating an uncompromised B to A, so KCI resistance is algebraically plausible; however, no reduction is supplied. Accordingly, KCI resistance, full CK/eCK AKE security, side-channel resistance, fault resistance, and secure erasure are not claimed.

Relation to ProVerif. The eleven true results establish only the secrecy and correspondence statements of the supplied symbolic Dolev–Yao model. The pairing computation is represented by an equation, and the primitives are perfect constructors/destructors; hence Table 3 does not prove the CDH assumption, random-oracle/KDF step, HMAC computational bound, invalid-point resistance, or the leakage cases in Table 4. The symbolic results and the computational sketch are complementary and must not be conflated.

Tool choice. ProVerif was selected because it automates secrecy and injective-correspondence analysis over unbounded concurrent sessions and directly supports the event-based authentication and replay properties modeled here. CryptoVerif targets game-based computational reductions but would require a separate probabilistic model of the exact pairing variant; it would not convert the present equational model into such a reduction. Tamarin supports mutable state and custom algebraic theories but would require a different model and additional lemmas for this workflow. Verifpal, Scyther, and AVISPA support rapid or bounded symbolic protocol analysis, but they do not by themselves analyze the construction’s exact pairing arithmetic or replace the computational argument. ProVerif is therefore used only for the stated symbolic scope, with the other tools viewed as possible complementary analyses.

## 5. Simulation Testing and Performance Evaluation

This section uses a multi-node software prototype to test handshake establishment, key confirmation, and data protection and to measure session establishment, data-plane processing, communication overhead, multi-ECU scalability, resource consumption, and security mechanism effectiveness. The processes represent the intended deployment roles; all timing measurements remain host-side.

### 5.1. Experimental Environment and Evaluation Metrics

**Figure 3:**
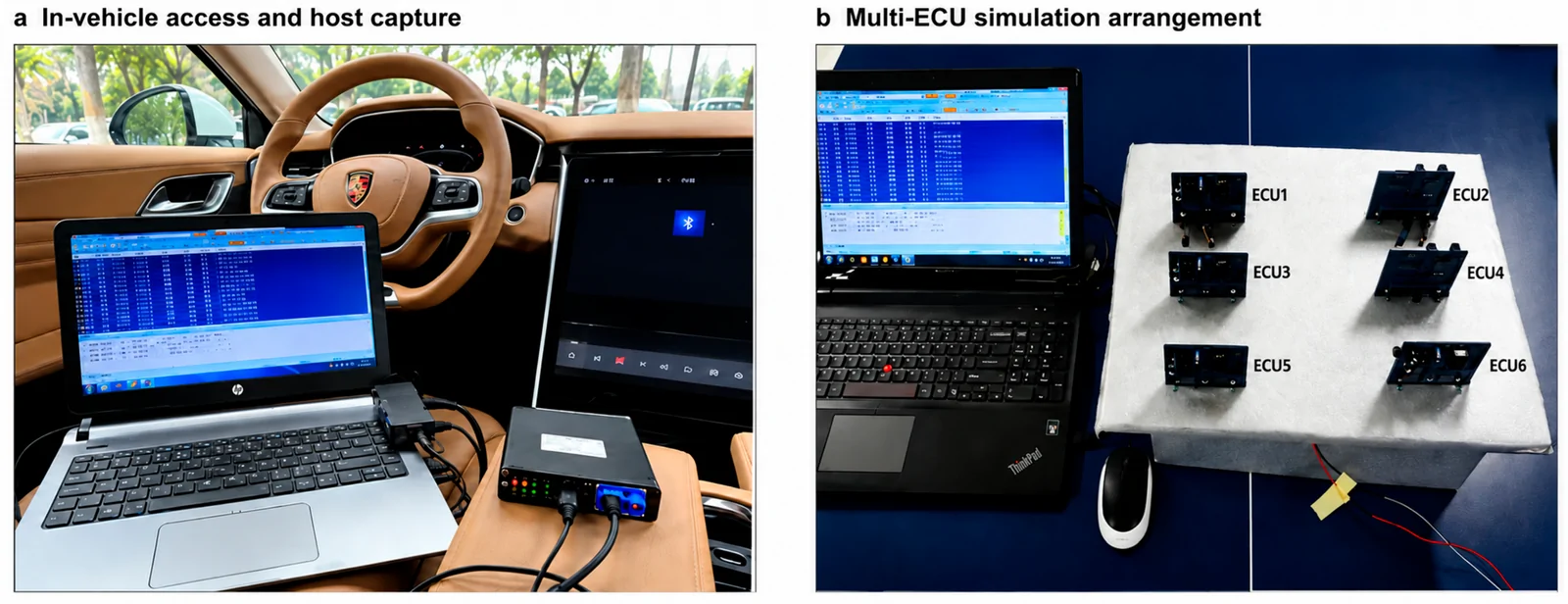
Experimental environment and multi-ECU arrangement: (**a**) in-vehicle access and host-side capture setup; (**b**) multi-ECU simulation arrangement. Non-English characters visible on the photographed screens belong to the original capture-software and vehicle infotainment interfaces, rather than experimental annotations. The component and panel labels are in English. The photographs document the arrangement; the reported latency results remain host-side measurements.

A multi-node software prototype was implemented to evaluate the proposed identity-based secure communication scheme for in-vehicle networks. The T-Box, gateway, and ECU nodes were implemented as independent software processes communicating through UDP sockets. Figure 3 illustrates the corresponding deployment roles and multi-ECU configuration. Unless otherwise specified, all reported latency results were obtained from host-side software measurements on this platform and therefore exclude physical CAN transmission, arbitration, controller queuing, and hardware-acceleration effects.

The evaluation covers session-establishment performance, data-plane processing latency, communication overhead, multi-ECU scalability, and security effectiveness. Session establishment was evaluated using end-to-end UDP completion time and the execution time of the principal cryptographic operations. For the data-plane evaluation, 30 independent sessions were established, and 240 fixed-length 8-byte application messages were processed under each parameter configuration. Scalability was evaluated for configurations containing 1, 4, 8, 12, and 18 ECUs, with three independent runs for each configuration. Communication overhead was analytically estimated using a 500-kbit/s classical-CAN model, excluding arbitration delay, dynamic bit stuffing, retransmissions, and transport-layer headers. Security effectiveness was evaluated using 12 frame-mutation attack classes and six packet-loss scenarios. The recorded metrics included rejection rate, receiver-side processing latency, plaintext-release events, loss-detection rate, and authenticated resynchronization performance.

#### Evidence Provenance and Hardware-Transfer Boundary

All latency values generated by this study are host-side observations from Windows/Python processes communicating through UDP or from a host-side function harness. The 287.560 ms handshake value includes protocol processing, serialization, process scheduling, and UDP communication on the prototype host. The 201.473 ms value is the SM9 session-key computation component on that host. The 1.036 ms value is the paired sender-plus-receiver processing median for one 8-byte message and excludes operating-system socket latency and physical CAN transmission. These values are prototype references, not embedded execution times or upper or lower bounds for a T-Box or ECU.

The 111-bit no-security baseline, 138.75 bits/message, 2.775% per ECU, and 49.95% at 18 ECUs are analytical outputs under a 500-kbit/s classical-CAN model, 100 application messages/s per ECU, an unstuffed 111-bit frame, ten frames per eight protected messages, and no retransmission. They are not measurements of bus occupancy. Replacing 111 with a conservative 135-bit frame-length sensitivity yields 60.75% for the same 18-ECU traffic point; this remains an analytical bound rather than a real-bus measurement.

The evaluation excludes arbitration-induced response time, dynamic bit-stuffing traces, controller queues, error frames and retransmissions, gateway buffering, hardware interrupts or DMA, driver and RTOS scheduling, clock drift, transceiver behavior, or physical-layer faults. On target hardware, end-to-end latency comprises target-specific cryptographic and software processing plus network and interface delays. Because processor, memory, implementation language, acceleration, driver, traffic priority, and bus conditions differ across platforms, no universal scaling factor or numerical hardware-transfer bound is claimed.

Only same-platform evidence supports a direct quantitative ranking. No such peer baseline is available in this study; the subsequent cross-scheme comparisons are therefore interpreted as analytical context rather than same-platform rankings. Appendix A Table A1 defines the evidence categories used in these comparisons.

### 5.2. Session-Establishment Performance Evaluation

Session-establishment performance was evaluated using single-ECU handshake latency and multi-ECU scalability. In the single-ECU experiment, a persistent T-Box process communicated with a persistent ECU process over UDP. Following a warm-up phase, 30 handshake trials were conducted, all of which completed successfully. As shown in Table 5, the median end-to-end T-Box completion time was 287.560 ms, the P95 latency was 292.545 ms, and the handshake success rate was 100%. The measured T-Box-side processing components included 32.509 ms for ephemeral-parameter generation, 201.473 ms for SM9 session-key computation, 5.619 ms for subkey derivation, and 7.494 ms for bidirectional key-confirmation generation and verification. The remaining latency was attributable to message serialization, process scheduling, UDP communication, and other protocol-processing operations. Among the measured cryptographic components, SM9 session-key computation accounted for the largest proportion of the session-establishment latency.

The 287.560 ms completion time and 201.473 ms SM9 component reported in Table 5 are Windows/Python/UDP host measurements. They exclude CAN/CAN-FD controllers, transceivers, arbitration, queues, retransmissions, interrupts, and target-ECU execution.

The multi-ECU experiment established sessions sequentially between one T-Box and 1, 4, 8, 12, or 18 ECUs. Three independent runs were conducted for each configuration and all tested establishments completed. As summarized in Table 6, total authentication time increased approximately in proportion to the ECU count over the tested range, while normalized authentication time remained between approximately 475 and 500 ms per ECU. For 18 ECUs, the median offline key-generation time was 405.416 ms, and the median sequential session-establishment time was 8551.829 ms, corresponding to 475.102 ms per ECU. These values describe the sequential Windows/Python/UDP prototype, not target-ECU timing bounds.

The results indicate that SM9 session-key computation was the primary contributor to session-establishment latency. Both the single-ECU test and the sequential multi-ECU tests achieved a 100% session-establishment success rate, while the total authentication time increased approximately linearly with the number of ECUs. In an embedded deployment, hardware acceleration, pairing precomputation, and parallel session scheduling may reduce the initial connection latency.

### 5.3. Data-Plane Processing Performance

Data-plane performance was evaluated by varying the authentication batch size k and transmitted tag length t for a fixed 8-byte application payload. For each configuration listed in Table 7, 30 independent SM9 sessions were established, and 240 messages were processed within each session. To reduce potential ordering bias, the configuration sequence was randomized within each session using the fixed pseudorandom seed 20260720. The reported host-side processing times include cryptographic computation, message serialization, and protocol processing but exclude physical CAN transmission, arbitration, controller queuing, and operating-system socket latency.

The 1.036 ms value reported in Table 7 is a paired sender-plus-receiver host-processing median for an 8-byte message. It excludes operating-system socket latency, controller queues, interrupts, arbitration, physical transmission, and retransmission.

**Figure 4:**
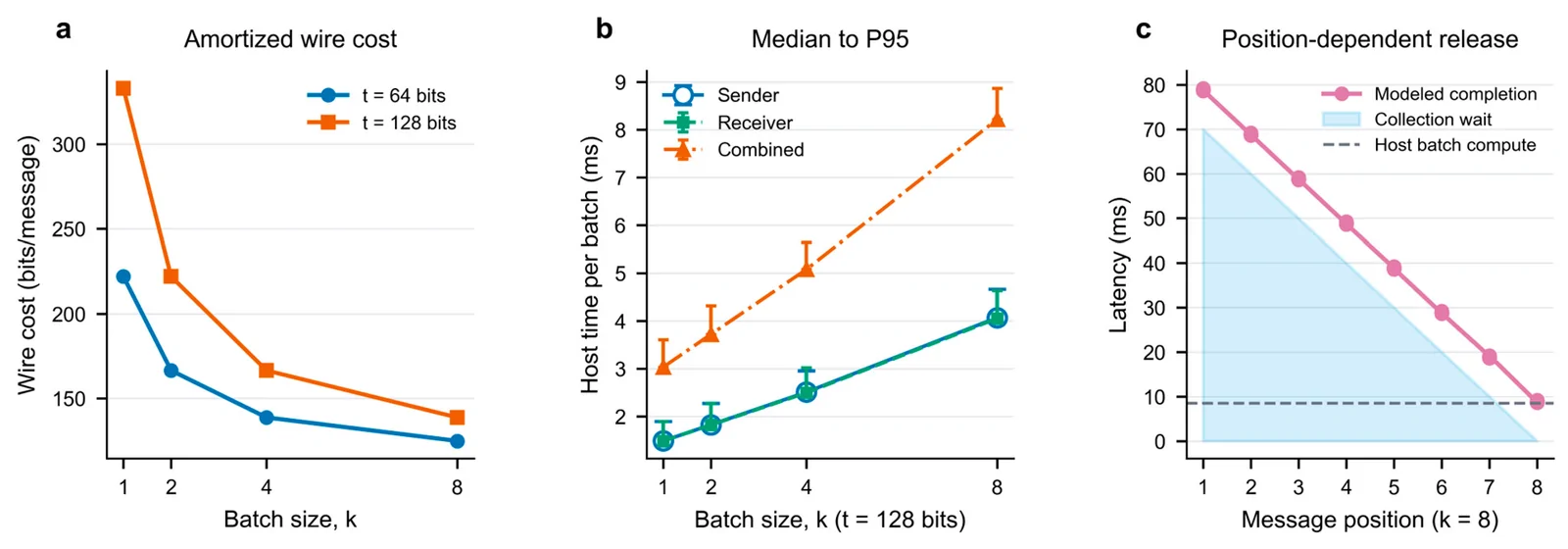
Compact-authentication trade-offs: (**a**) amortized wire cost versus batch size for 64-bit and 128-bit tags; (**b**) sender, receiver, and combined host processing time per batch for 128-bit tags, with markers indicating medians and upper whiskers extending to P95; (**c**) position-dependent modeled release completion for an eight-message batch, showing collection waiting time and the measured host batch-computation component. In (**b**), blue hollow circles and smaller green squares distinguish the nearly coincident sender and receiver series without changing their values.

For the configuration of k = 8 and t = 128, the sender-side and receiver-side medians were 0.521 and 0.516 ms/message, respectively. The median of the paired sender-plus-receiver totals was 1.036 ms/message, with a P95 of 1.074 ms/message. Increasing the batch size from k = 1 to k = 8 reduced the combined median processing time from 3.108 to approximately 1.038 ms/message by amortizing the costs of HMAC generation, verification, message serialization, and related protocol processing across multiple messages. When k = 8, increasing the tag length from 64 to 128 bits changed the combined median only from 1.038 to 1.036 ms/message under the host-side test conditions used here while increasing the average frame cost from 1.125 to 1.250 frames/message. Considering the trade-off between processing latency, communication overhead, and authentication strength, the k = 8 and t = 128 configuration was selected for the subsequent evaluation. Figure 4 summarizes the effects of batch size and tag length on amortized wire cost, host processing time, and position-dependent message release.

### 5.4. Communication Overhead and Bus Load Analysis

**Figure 5:**
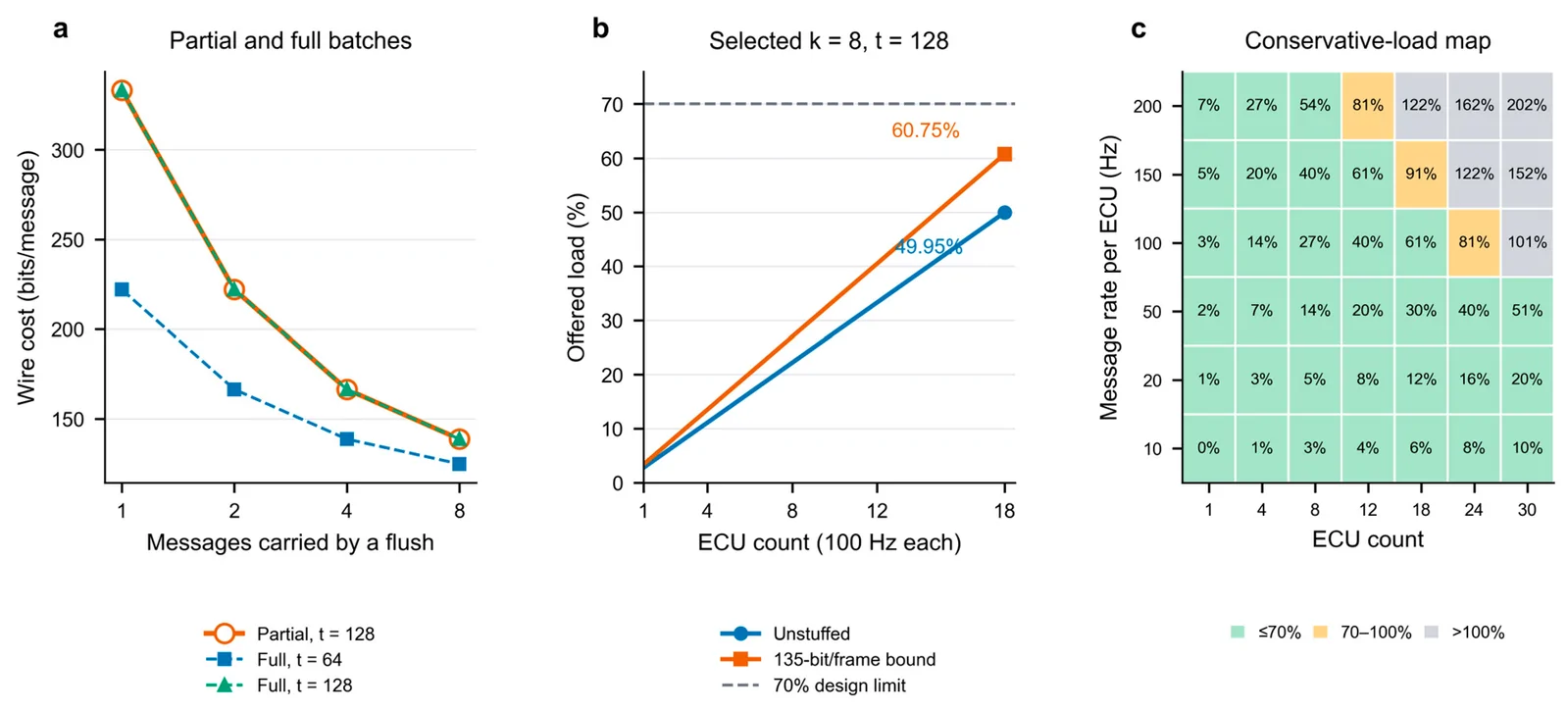
Compact-authentication bus cost and analytical operating region: (**a**) amortized wire cost for partial flushes with 128-bit tags and full batches with 64-bit or 128-bit tags; (**b**) offered load versus ECU count for k = 8, t = 128 bits, and 100 messages/s per ECU, comparing the unstuffed model with the conservative 135-bit/frame bound; (**c**) conservative offered-load map versus ECU count and message rate per ECU. In (**a**), the 128-bit partial-flush and full-batch curves coincide where their batch sizes match; different markers identify both series. Green, yellow, and gray cells in (**c**) indicate loads at or below 70%, above 70% and at or below 100%, and above 100%, respectively.

In the full-batch configuration, eight DLC-8 data frames carry eight independently encrypted 8-byte application messages, while two additional DLC-8 frames carry the 128-bit HMAC-SM3 authentication tag. A complete batch therefore comprises ten CAN frames with a total payload of 80 bytes, corresponding to an average of 10 bytes and 1.25 frames per application message. Routing information and frame type are encoded in preconfigured CAN identifiers, whereas a receiver-maintained batch sequence number provides freshness protection. Both the identifier-related information and the sequence number are incorporated into the authenticated context. For a partial batch containing m application messages, the sender transmits m data frames and two authentication frames. Consequently, under the unstuffed classical-CAN frame model, the amortized communication cost decreases from 333 bits/message for m = 1 to 138.75 bits/message for m = 8. Figure 5 presents the wire-cost and analytical bus-load trade-offs for partial and full batches and the selected k = 8, t = 128-bit profile.

For the 18-ECU configuration, the common analytical model yields an offered load of 49.95% for the proposed profile, 47.16% for the WSP lower-bound mapping, 62.64% for LEAP, 73.80% for EAS, 79.92% for MAuth-CAN, and 59.94% for the Sensors 2025 mapping. These values follow from the assumptions in Table 8; they are not measurements of the cited implementations. EC-SVC is not included because it targets CAN FD and uses an edge-based security agent [30].

The percentages in Table 8 are arithmetic mappings under the stated evidence boundaries rather than same-platform measurements. No peer scheme was reimplemented on the Windows/Python host; consequently, the values do not establish lower real-bus utilization, lower embedded latency, or superior overall performance.

Appendix A Table A2 lists the evidence sources and mapping assumptions. The Sensors row uses 1.50 × 111 = 166.50 bits/message, consistent with 13.32% at four ECUs and 59.94% at eighteen ECUs. Percentage differences are within-model arithmetic and are not measured performance advantages.

**Figure 6:**
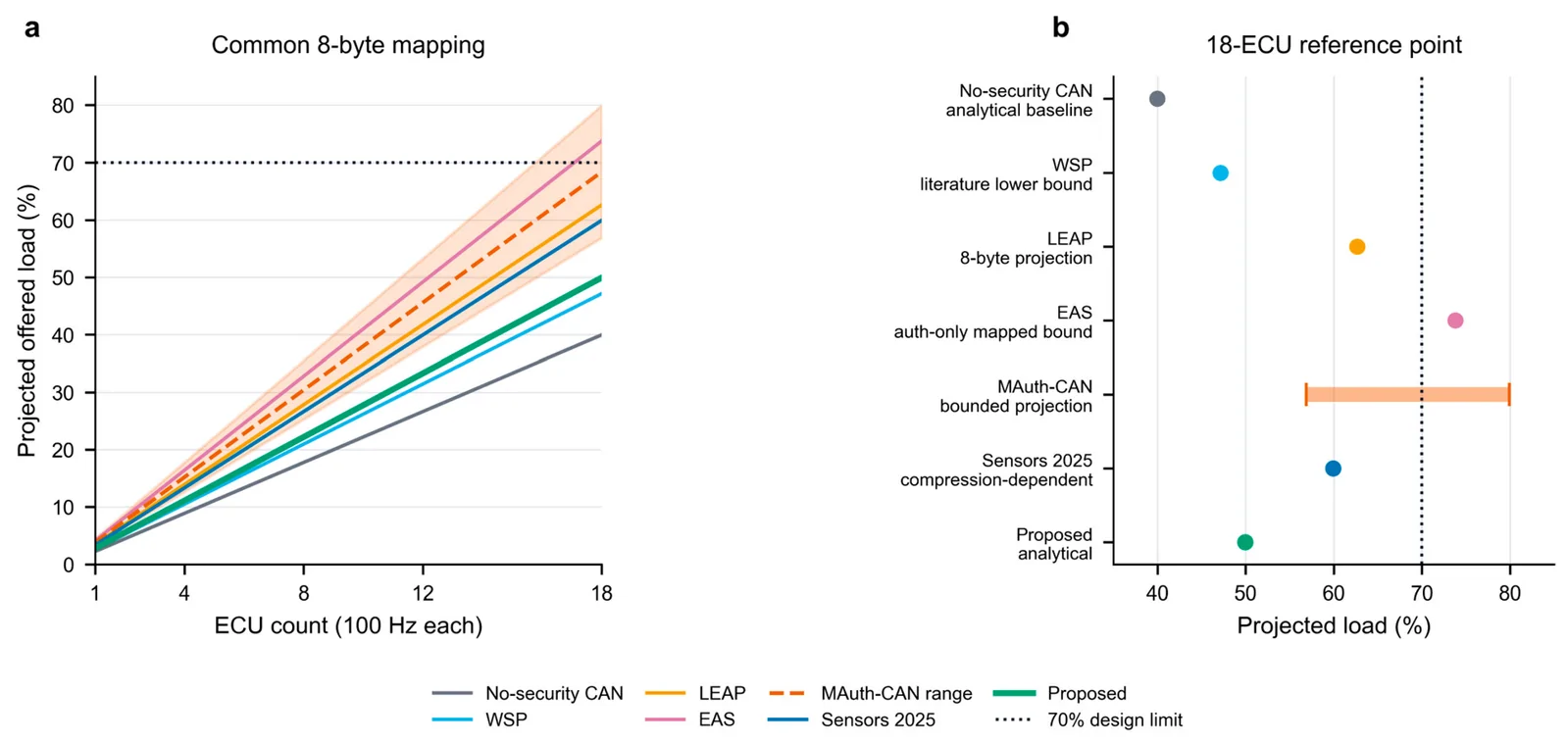
Frame-cost context under a common 8-byte analytical mapping: (**a**) projected offered load versus ECU count at 100 messages/s per ECU; (**b**) projected load at 18 ECUs, with each reference identified by its evidence class. The literature context comprises WSP [34], LEAP [32], EAS [35], MAuth-CAN [31], and the Sensors 2025 study [36]. Colors identify the schemes in the shared legend; the orange band in (**a**) and interval in (**b**) show the MAuth-CAN range. Dotted lines mark the 70% design limit. These mapped or bounded reference values do not constitute same-platform performance rankings.

**Figure 7:**
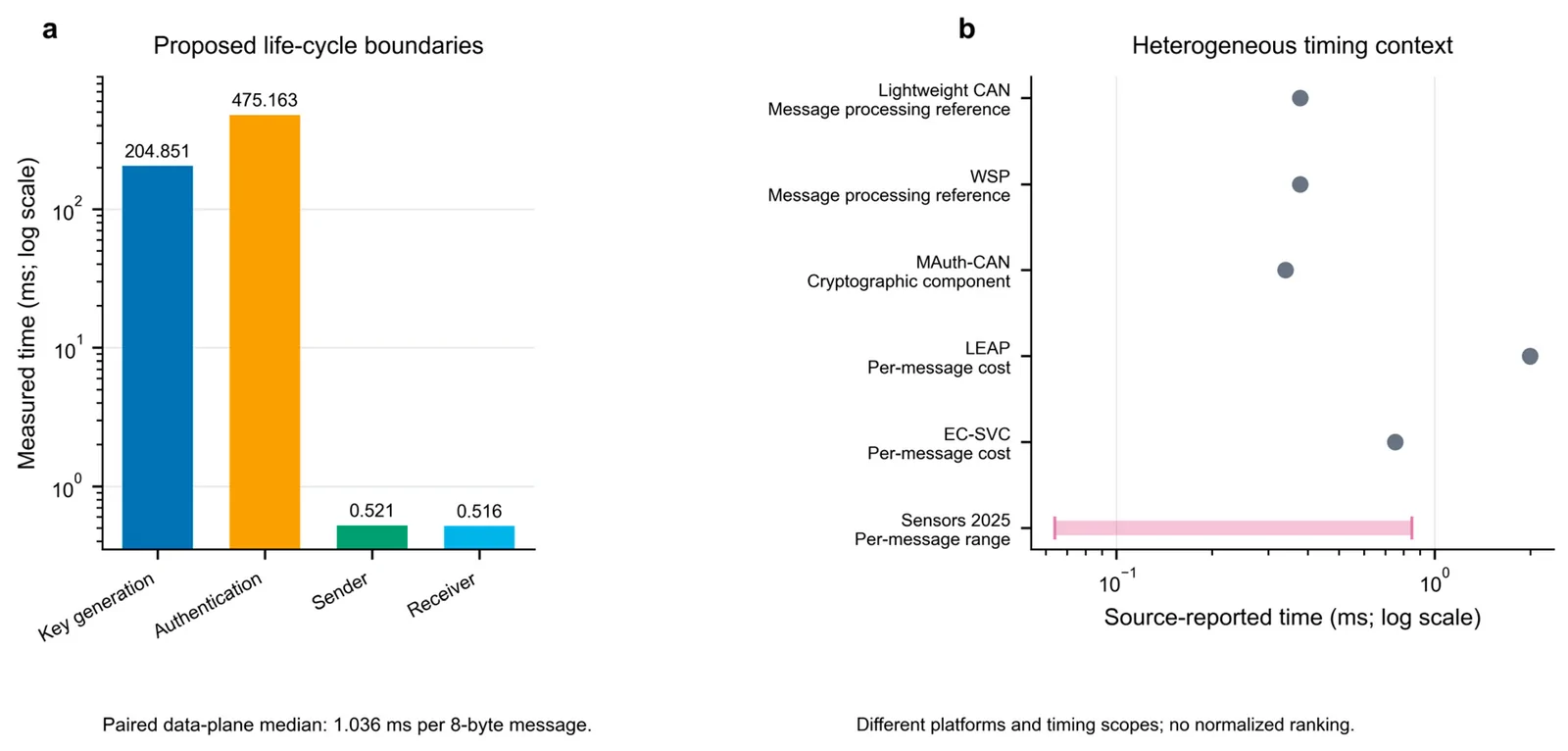
Life-cycle timing boundaries and heterogeneous literature context: (**a**) host-measured offline key generation, online authentication, sender processing, and receiver processing for the proposed implementation; (**b**) source-reported timing references for Lightweight CAN [37], WSP [34], MAuth-CAN [31], LEAP [32], EC-SVC [30], and the Sensors 2025 study [36]. In (**a**), blue, orange, green, and light blue identify the four operations, respectively. The paired sender-plus-receiver data-plane median is 1.036 ms per 8-byte message and is not the sum of the two marginal medians. In (**b**), gray points show individual reported values and the pink interval shows a reported range. Both panels use logarithmic time axes. The literature references differ in platform and measurement scope and do not provide a normalized performance ranking.

Figure 6 and Figure 7 visualize analytical and literature-derived mappings. They are not traces from a physical CAN/CAN-FD bus and do not represent peer implementations executed under a common platform or workload.

### 5.5. Life-Cycle Time Decomposition and Qualified Cross-Scheme Comparison

To clarify the measurement boundaries, life-cycle cost is decomposed into offline key generation, online authentication, and established-session data processing. T_KG_ includes KGC setup and extraction of the identity keys for the T-Box and target ECU. T_AUTH_ includes both ephemeral generations, both SM9 session-key computations, directional KDF operations, bidirectional key-confirmation generation and verification, and auxiliary serialization. For the 8-B data plane, T_S,opt_ and T_R,opt_ denote sender protection and receiver verification/decryption, respectively. Table 9 summarizes the life-cycle time decomposition and the corresponding measurement boundaries.

(42) $$T_{\mathrm{total},\mathrm{cold}}^{\mathrm{ref}}=T_{\mathrm{KG}}+T_{\mathrm{AUTH}}+T_{S,\mathrm{opt}}+T_{R,\mathrm{opt}}$$

*T*_KG_ = *T*_setup_ + *T*_ext,T_ + *T*_ext,E_ (43)

*T*_AUTH_ = *T*_R_ + *T*_KA_ + *T*_KDF_ + *T*_KC_ + *T*_aux_ (44)

*T*_DATA,opt_ = *T*_S,opt_ + *T*_R,opt_ (45)

The 681.050 ms cold and 476.199 ms online references sum marginal medians from separate life-cycle and compact 8-B data-plane experiments. Each sum only estimates a combined median; it is not a directly measured median of paired totals or an end-to-end wall time. In contrast, paired sender-plus-receiver values yield the measured data-plane median of *T*_DATA,opt_ = 1.036 ms/message.

The proposed value and the 1-ECU value in Table 6 come from different experiment blocks and are not duplicate measurements. A blank, unresolved, or differently scoped peer component is not treated as zero. Appendix A Table A3 lists the timing components and source-reported reference points. Appendix A Table A4 records the evidence categories and comparability limits of the timing data.

Figure 7 compares timing values reported under different processors, payloads, algorithms, and measurement scopes. Their visual proximity does not imply an aligned benchmark or an ordering of implementation performance.

### 5.6. Attack-Frame Mutation, Rejection, and Authenticated Resynchronization

The attack experiment mutates the compact frames used during normal traffic. For each case, the test harness records the trial identifier, independent session block, representative frame mutation, receiver rejection reason, processing latency, and number of plaintext messages released. Each of the 12 attack classes was tested 100 times. A timeout or explicit state violation is counted as a rejection only when no unauthenticated plaintext is delivered. Table 10 summarizes the receiver outcomes for the twelve representative frame-mutation attacks.

All 1200 mutated-frame trials were rejected, and no attacked batch released plaintext. Ciphertext, tag, ordering, and replay mutations failed authentication; inserted frames and data/authentication identifier confusion were rejected by the batch state machine; unknown identifiers failed route lookup; truncated authentication fragments were detected at timeout; and a 64-bit receiver interpretation of a 128-bit sender profile failed because the tag length was included in the authenticated context. These finite tests exercise the implemented rejection paths; they neither constitute a formal proof nor demonstrate availability under sustained flooding. Table 11 reports loss detection and authenticated counter-resynchronization results.

For the loss of D1, D4, D8, A1, A2, or two data frames, every damaged batch was detected without plaintext release. In all 30 trials per scenario, the next complete batch was accepted after authenticated resynchronization. Recovery requires three frames, corresponding to a 333-bit unstuffed lower bound, and the measured host-processing median ranges from 2.327 to 2.406 ms. This exceptional control traffic is excluded from the steady-state 2.775% per-ECU load estimate.

#### Adverse Loss and Counter-Desynchronization Evaluation

The authenticated-resynchronization experiment was extended from isolated frame-loss cases to 1, 8, 64, 256, 1024, 4096, and 16,384 consecutively lost application messages. Each level was repeated five times with batch size 8 and a 128-bit authentication tag. All data and tag frames generated during the loss interval were dropped; the sender then emitted the three-frame authenticated resynchronization message, after which the next complete eight-message batch was transmitted and checked. Times below measure only host-side verification and state application of the three resynchronization frames.

All 35 trials recovered the receiver counter and accepted the next complete batch, with zero unauthenticated plaintext releases. The median resynchronization-state processing time remained between 1.0983 and 1.3178 ms across the tested loss levels because the authenticated control message has a fixed three-frame format; this timing excludes the duration of the loss interval, CAN arbitration, retransmission, controller queuing, and the time needed to deliver the control frames. The trials characterize recovery after communication resumes; they do not evaluate sustained jamming, bus-off, or repeated loss of the resynchronization message. Appendix A Table A5 reports authenticated resynchronization under sustained message loss.

### 5.7. Cross-Scheme Trade-Offs and Security Boundaries

The comparison does not identify a single scheme as preferable across all communication, computation, and security dimensions. Under the common 8-B classical-CAN model, the proposed profile requires 138.75 bit/message and produces 49.95% offered load at 18 ECUs. The projected differences relative to WSP, LEAP, EAS, MAuth-CAN, and the Sensors 2025 mapping are model-based comparisons under the stated assumptions, not same-platform performance measurements. Table 12 summarizes the qualified cross-scheme comparisons of the proposed compact data plane.

The 20.26%, 32.32%, and 12.18–37.50% differences describe projected bits per 8-byte application message within the stated mapping. They are not measured performance advantages. In the absence of a same-platform peer baseline, the comparison is reported as a boundary-qualified trade-off analysis.

Appendix A Table A6 reports the analytical differences using the stated mapping. A negative value denotes fewer modeled bits under those assumptions; it does not establish faster execution, lower measured utilization, stronger security, or superior deployment.

The measured data-plane median of the proposed implementation is 1.036 ms per 8-B message on the Windows host. Published values such as 0.064 ms for Sensors 2025 at 400 MHz, 0.753 ms for EC-SVC with a 48-B CAN-FD payload, 1.998 ms/message for LEAP on a 16-MHz ATmega328, and 5.153 ms for the Lightweight CAN encryption reference use different processors, payloads, algorithms, and timing boundaries [30,32,37]. They provide engineering context but do not support a platform-independent speed ranking. Within the present implementation, T_AUTH_ = 475.163 ms dominates the established-session data path, whereas the offline T_KG_ = 204.851 ms cost can be amortized over the key lifetime.

**Figure 8:**
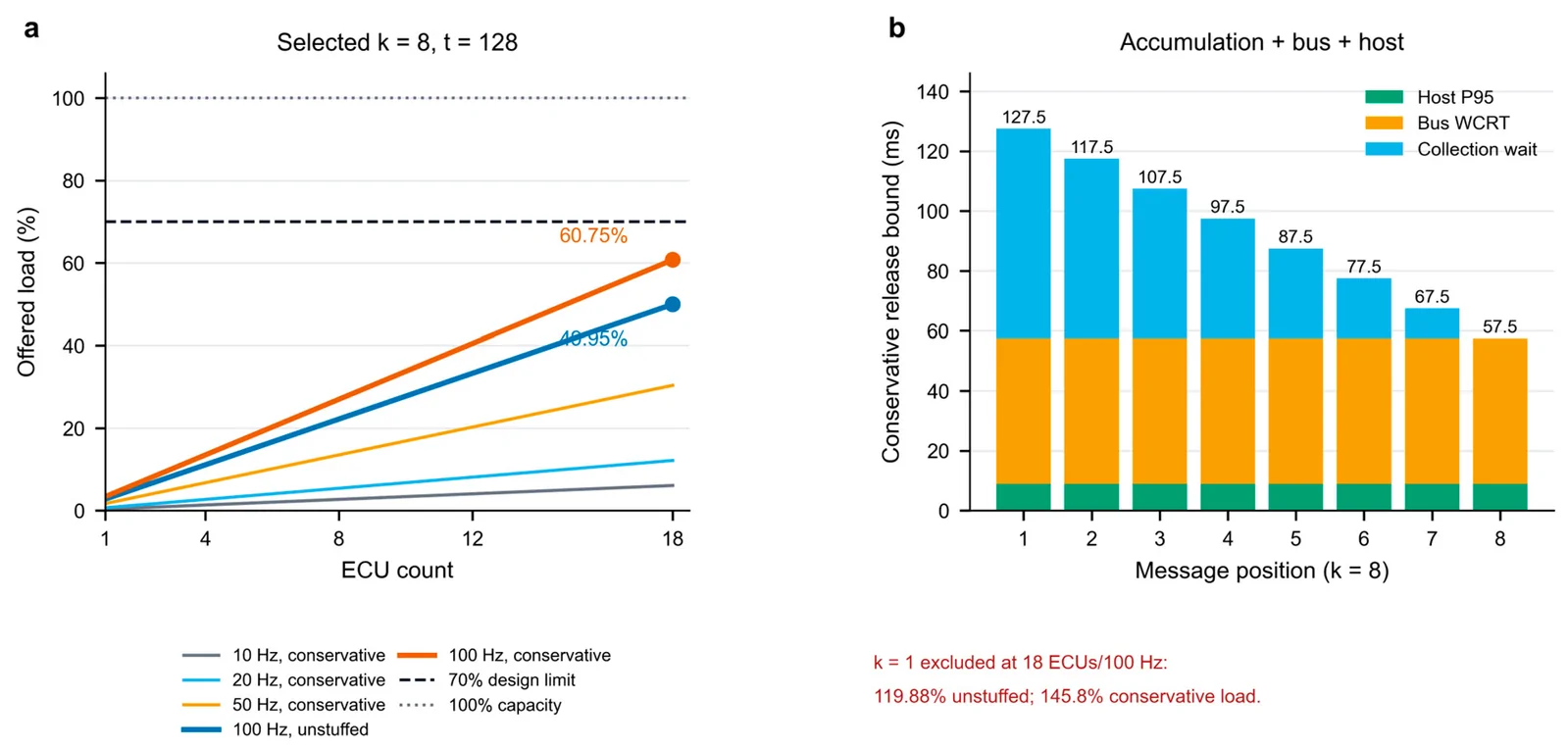
Selected k = 8, t = 128-bit operating profile: (**a**) offered load versus ECU count under different message rates, including the unstuffed 100-Hz case and conservative bit-stuffing bounds, with the 70% design limit and 100% capacity shown separately; (**b**) conservative release bound versus message position within an eight-message batch, decomposed into collection waiting time, bus worst-case response time, and measured host-processing P95. In (**b**), light blue, orange, and green denote these three components, respectively. The bounds combine analytical bus response with host measurements and do not represent measured target-ECU end-to-end latency.

The proposed profile combines confidentiality, identity-bound session establishment, directional key separation, integrity and replay protection, route and policy binding, standard CAN-frame compatibility, and verify-before-release processing. One 128-bit tag covers eight messages. At 18 ECUs and 100 Hz, the position-dependent release bounds for the first, average-position, and last messages are 127.4684, 92.4684, and 57.4684 ms, respectively. Frame loss can trigger a three-frame authenticated resynchronization. The profile targets cyclic status, monitoring, and diagnostic traffic that tolerates aggregation; hard-real-time braking, steering, or restraint-control traffic requires a shorter batch, per-message authentication, or CAN FD. Sustained denial-of-service, arbitration monopolization, and bus-off behavior remain outside the evaluation. Table 13 summarizes the security functions and deployment boundaries of the compared schemes. Figure 8 presents the offered load and position-dependent conservative release bound for the selected k = 8, t = 128-bit profile.

### 5.8. Batch Accumulation, CAN Response Bound, and Security Trade-Offs

The 49.95% value is the offered load obtained from a 111-bit unstuffed standard 8-byte classical-CAN frame. The corresponding conservative bit-stuffing upper bound is 60.75%; the 98-bit stuffable region permits at most floor((98 − 1)/4) = 24 additional bits, giving 135 bits per frame. A release bound must include multi-ECU queueing and is therefore evaluated with the batch-response model below.

The analysis separates (i) batch accumulation, (ii) measured host cryptographic processing, and (iii) classical-CAN transmission response. For N ECUs transmitting at f messages/s with batch size k and a t-bit tag, a = t/64 authentication frames are appended per batch. Thus, frames/message = 1 + a/k, *T_batch_* = 1000*k*/*f*ms, and *C_batch_* = 1000(*k* + *a*)135/500,000 ms. A synchronous fixed-priority batch-burst model reports the lowest-priority ECU response. The 100% capacity test is kept separate from a 70% engineering-headroom screening line; the latter is a design choice, not a standards guarantee.

Each profile used 20 independent session blocks and 160 fixed 8-byte messages per block, with a fixed-seed randomized profile order. P95 is calculated across complete batches. The timed path comprises the copied sender and verify-before-release receiver; it excludes the SM9 handshake, bus I/O, and target-ECU WCET. Every released batch matched its input, and no plaintext was released before the complete authenticator arrived. Table 14 summarizes load, schedulability, and authentication-release bounds for the evaluated batch sizes. Appendix A Table A7 and Table A8 provide the complete-batch processing times and message-rate sensitivity results, respectively.

For k = 8, the copied implementation’s sender-plus-receiver batch-processing P95 is 8.8684 ms, and the synchronous batch-burst bus-response bound is 48.6000 ms. Adding the 70/35/0 ms position-dependent accumulation waits gives 127.4684/92.4684/57.4684 ms for the first-produced, average-position, and last-produced messages.

Lower message rates reduce bus load but do not necessarily make large batches compatible with latency requirements. At 10 Hz, k = 8 imposes a 700 ms accumulation wait on the first-produced message. Low-rate or deadline-constrained streams therefore require a smaller batch, an authenticated partial-batch timeout, or per-message authentication.

Under the ideal truncated-MAC model, the per-attempt guessing probability is 2^−*t*^. Increasing k does not increase the tag’s bit strength: it reduces the number of verification opportunities and amortizes authentication frames, but one accepted forged batch can affect up to k messages and the accumulation delay increases. These bounds assume an uncompromised key and independent online guesses; they do not cover key exposure, implementation flaws, side channels, or denial of service. Appendix A Table A9 lists the ideal online-forgery bounds under these assumptions.

The k = 8 profile is intended for monitoring, status reporting, and non-critical diagnostic traffic whose end-to-end deadline exceeds the scenario-specific release bound. It is not evaluated for hard-real-time braking, steering, or occupant-protection traffic. Such flows require per-message protection or a smaller batch, hardware-target worst-case execution-time measurements, the actual CAN-ID priority set, interference from all traffic, fault and retransmission analysis, and vehicle-level timing validation.

### 5.9. SM9 Establishment Bottleneck and Multi ECU Scalability

In the July independent-process UDP experiment, the T-Box generate_session_key call had a mean duration of 201.473 ms; this component is distinct from the complete 287.560 ms handshake and from target-ECU hardware execution. A separate 30-run operation profile measured a 205.371 ms mean and decomposed it into the variable-input pairing e(R_peer_,D_self_), fixed-base exponentiation g^x^, pairing-result exponentiation $g_{1}^{x}$, and encoding/hash/KDF work. The two values come from separate experiment blocks and are reported separately.

Each scale and mode uses thirty timed session blocks after two excluded warm-up blocks. Every timed session generates fresh ephemeral scalars, public values, and session identifiers, while persistent endpoint processes and provisioned long-term keys keep process creation, socket binding, and offline KGC work outside the online boundary. The 1/4/8/12/18-ECU points represent a single on-demand session, a small functional domain, two staged OTA cohorts, and the upper tested synchronized burst; they are sensitivity points rather than a universal vehicle topology claim. Table 15 presents the operation-level profile of T-Box session-key computation.

The variable-input pairing averages 177.461 ms and accounts for 86.41% of the measured session-key computation. The fixed pairing g = e(P_pub_,P_2_) is already calculated during offline setup and cached in the public parameters. The remaining online pairing depends on the fresh peer point R_peer_ and cannot be precomputed across sessions. The implemented safe variant caches the deterministic public identity point Q_ID_ and schedules the current session’s g^x^ after HELLO; x, R, and g^x^ remain fresh for every session. Byte-for-byte tests confirm that the baseline and precomputed paths derive identical 32-byte roots at both endpoints. Table 16 reports multi-ECU establishment makespans across the three execution modes.

At 18 ECUs, concurrent scheduling reduces the median critical-path makespan from 5368.785 to 4509.737 ms (1.190×); safe precomputation reduces it further to 4201.479 ms, or 21.74% below the sequential baseline. One ECU has no parallel work, so concurrent scheduling adds slight coordination overhead. The 8551.829 ms value is a separate three-run, one-process life-cycle measurement with sequential execution of both endpoints and is not used as the denominator for these speedups.

Total/ECU is a throughput-equivalent normalization rather than an observed per-session latency. In the 18-ECU concurrent burst, the normalized median is 250.541 ms, whereas the observed session-completion median is 4176.826 ms because all sessions become active before the single T-Box event loop serially processes their SM9 work. Sequential per-session variation (median 297.298 ms, SD 6.360 ms, and P95 309.341 ms) reflects OS scheduling, Python interpreter and memory behavior, UDP loopback queueing, and data-dependent large-integer and curve operations for random scalars.

**Figure 9:**
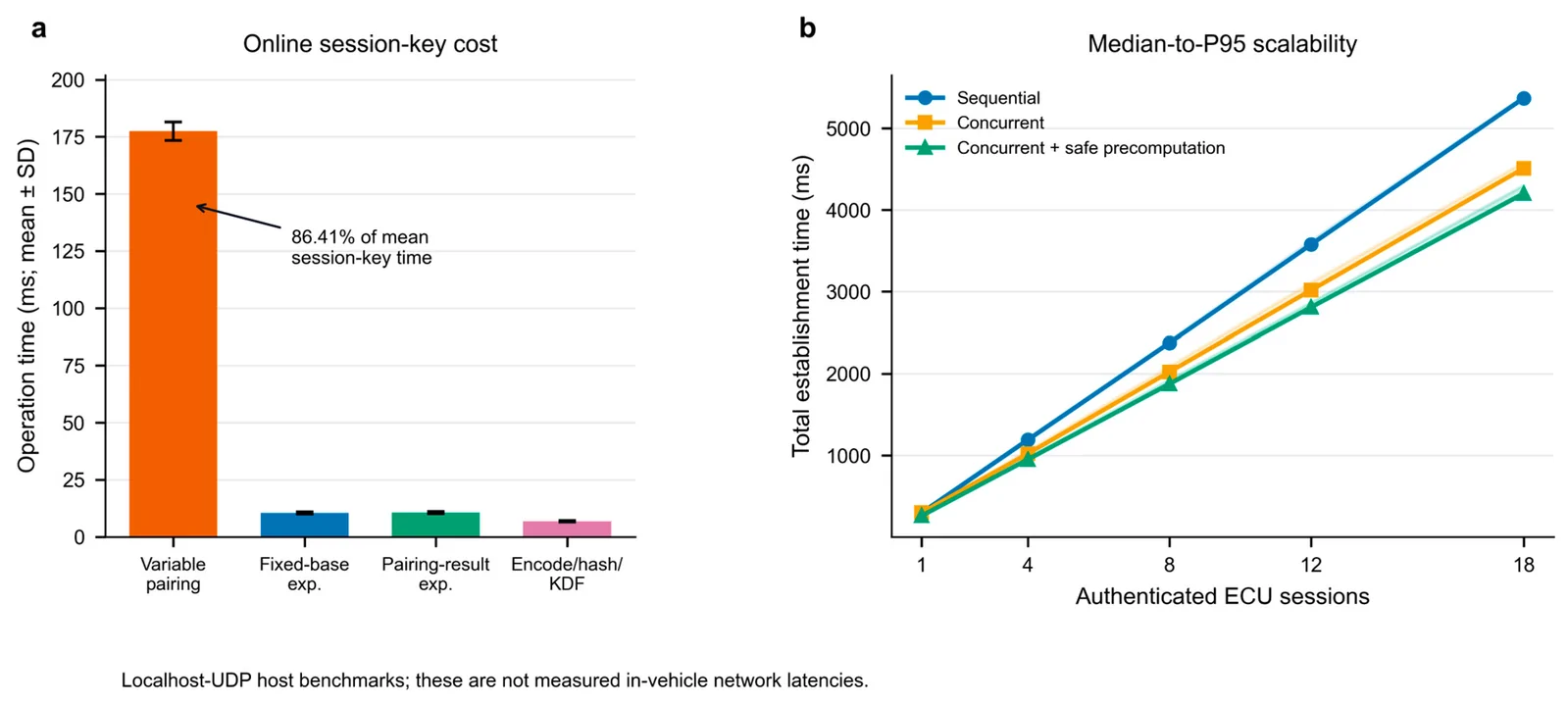
Online session-key computation and localhost-UDP scalability: (**a**) operation-level session-key cost, with bars showing means and error bars showing standard deviations over 30 runs; variable pairing accounts for 86.41% of the mean session-key computation time; (**b**) total multi-ECU establishment time versus the number of authenticated ECU sessions for sequential execution, concurrent execution, and concurrent execution with safe precomputation. Markers show medians and shaded envelopes extend to P95. In (**b**), blue, orange, and green identify these modes, respectively. These are host benchmarks, not measurements of in-vehicle network latency.

One-ECU on-demand diagnostics complete in a median 260.340 ms with the evaluated precomputation variant, whereas an 18-ECU synchronized startup or expiry-recovery burst requires a median 4201.479 ms. OTA and non-critical diagnostic sessions can therefore be established and reused before data transfer, with staged cohorts or staggered expiry where policy permits. These host-side values are not target-hardware or physical-bus timing bounds. Figure 9 shows the online session-key operation breakdown and localhost-UDP scalability results. Appendix A Table A10 and Table A11 report per-ECU completion-time variation and representative access patterns, respectively.

## 6. Limitations

The empirical timing results are specific to the Windows/Python/UDP prototype. The 287.560 ms single-ECU handshake includes host protocol processing and UDP communication, whereas the 1.036 ms per-message value measures paired sender and receiver processing for an 8-byte payload and excludes socket latency and bus I/O. Neither value is a target-ECU execution bound. The 138.75-bit message cost and 49.95% offered load are analytical outputs of the stated unstuffed 500-kbit/s classical-CAN model, not physical-bus measurements. Controller queuing, arbitration, bit stuffing, retransmissions, gateway buffering, interrupts, and RTOS scheduling require measurement on the intended hardware and network.

The selected batch size of eight and 128-bit tag amortize authentication overhead but delay plaintext release until the complete batch has been verified. At 18 ECUs and 100 messages/s per ECU, the scenario-specific first-message release bound is 127.4684 ms; lower message rates can further increase accumulation time. Thus, the evaluated profile is intended for monitoring, status reporting, and non-critical diagnostic traffic whose deadlines tolerate this delay. It has not been validated for hard-real-time braking, steering, or occupant-protection traffic, which would require smaller batches or per-message authentication and target-specific worst-case timing analysis.

The eleven true ProVerif queries establish the modeled secrecy and correspondence properties only under the Dolev–Yao and perfect-cryptography assumptions. They do not constitute a computational proof of the SM9-based pairing variant, whose BN254/alt_bn128-style backend has not undergone official GM/T 0044 known-answer or interoperability testing. The analysis assumes a trusted KGC and protected participating long-term identity keys. Full forward secrecy under broader key-compromise models, KCI resistance, full CK/eCK security, side-channel and fault resistance, and secure erasure are not established.

The frame-mutation and loss experiments exercise specified finite attack classes and recovery cases; they do not establish availability under sustained denial of service, jamming, arbitration monopolization, or bus-off behavior. The cross-scheme comparisons use analytical mappings and literature-reported values from different platforms and workloads because no peer scheme was reimplemented under the same conditions. Accordingly, they do not support a measured performance ranking.

## 7. Conclusions

This paper presents an identity-based secure communication scheme for in-vehicle networks. An SM9-based pairing variant is used for authenticated session establishment with explicit bidirectional key confirmation. Direction- and purpose-specific subkeys support SM4-CTR encryption and HMAC-SM3 authentication during established sessions. The framework maintains an isolated context for each T-Box–ECU session and specifies T-Box decrypt-and-forward via the gateway and end-to-end ciphertext-forwarding modes.

Under the assumptions of the ProVerif symbolic model, all eleven secrecy and injective-correspondence queries returned true. In the Windows/Python/UDP prototype, the median single-ECU handshake completion time was 287.560 ms. With a batch size of eight and a 128-bit authentication tag, the median combined sender-and-receiver processing time was 1.036 ms per 8-byte message. Under the adopted unstuffed 500-kbit/s classical-CAN model, this profile required 138.75 bits per message and yielded 49.95% offered load for 18 ECUs transmitting 100 messages per second each. All 1200 controlled frame-mutation trials were rejected without unauthorized plaintext release.

The selected profile is intended for monitoring, status reporting, and non-critical diagnostic traffic whose deadlines accommodate batch accumulation and authenticated release. These findings are limited to symbolic analysis, host-side measurements, controlled tests, and analytical bus-load estimates. Embedded-ECU timing, physical-bus behavior, and hard-real-time suitability remain unverified. Full forward secrecy under broader long-term-key compromise models has not been established by the present analysis.

## Figures and Tables

**Table 1:** Scope-based comparison with representative prior work (NR: not reported in the cited source).

Scheme	Authentication and Key Dependency	Replay/Freshness	Protected Scope and Forwarding	Evaluation Platform
Groza and Murvay [15]	Signed DH plus pairing and pairing-free identity-based variants; identity-derived public keys; pairwise and common session-key establishment.	Fresh shared keys and forward-secrecy goal discussed; application-data replay-window policy NR.	Key-exchange feasibility and common-key extension; T-Box per-ECU contexts and the two forwarding modes NR.	Automotive-grade controllers; CANoe; CAN-FD and FlexRay.
AUTOSAR SecOC [29]	PDU authenticity verification using configured cryptographic material and freshness management; no identity-based handshake in the SecOC module specification.	Freshness values, truncation profiles, and receiver verification are defined.	Secured I-PDU authenticity/freshness; T-Box dual forwarding architecture NR.	Industry specification; implementation-dependent.
EC-SVC [30]	Edge-assisted secure operations with fine-grained attribute-based access control.	Separate sequence-window policy NR in the public source summary.	Secure CAN communication through an edge device; the proposed two T-Box forwarding modes NR.	Formal proof and CANoe evaluation.
MAuth-CAN [31]	Authenticator-centered CAN message authentication targeting masquerade attacks; no identity-based key establishment in the reported scope.	Explicit sliding-window policy NR in the public abstract.	Message authentication and bus-off mitigation; confidentiality and T-Box dual forwarding NR.	Protocol analysis/evaluation reported; not a common platform with this work.
LEAP [32]	Security-enhanced stream-cipher-based CAN encryption and authentication; symmetric-key dependency.	Separate replay-window policy NR in the public abstract.	CAN message data plane; identity-based setup and T-Box dual forwarding NR.	Embedded/software performance evidence; not a common platform with this work.
Proposed scheme	SM9 identity-bound setup with KGC-provisioned private keys; explicit confirmation; SM4/HMAC subkeys for established sessions.	Per-direction sequence numbers, session binding, and rejection before plaintext release; operational window policy stated separately.	Confidentiality, authentication, and replay protection; per-ECU T-Box contexts; decrypt-and-forward and ciphertext-forwarding modes.	ProVerif; Windows/Python/UDP host tests; analytical classical-CAN model; no hardware validation.

**Table 2:** Consolidated notation and parameter definitions.

Symbol	Definition and Use
params	Published SM9 system parameters, including groups, generators, pairing, hashes, and encodings.
G_1_, G_2_, G_T_	Source groups and target group of prime order *n*.
P_1_, P_2_	Generators of G_1_ and G_2_, respectively.
*n*	Prime group order; arithmetic on private scalars is performed modulo *n*.
e	Bilinear pairing e: G_1_ × G_2_ → G_T_.
*s*/*msk*	KGC master private scalar in $Z_{n}^{*}.$
*P*_pub_/*m*_pk_	Master public key *P*_pub_ = *s*P_1_.
ID_A_, ID_B_	Identities of T-Box A and target ECU B.
H_1_	Hash-to-integer function $H_{1}:{0,1}^{*}\to Z_{n}^{*}.$
*tag*_ID_	Public function-domain label used only during identity-key extraction.
h_U_, Q_U_	Identity hash h_U_ and public identity point Q_U_ = (h_U_ + *s*)P_1_ for U in {A,B}.
*t*_1_, *k*_i_, *D*_i_	Private-key extraction intermediate, scalar, and long-term identity private point.
*r*_A_, *r*_B_	Fresh one-session random scalars selected by A and B.
*R*_A_, *R*_B_	Ephemeral negotiation points *R*_A_ = *r*_A_Q_B_ and *R*_B_ = *r*_B_Q_A_.
g	Base pairing value e(*P*_1_,*P*_2_)*^s^* in G_T_ used only to explain equality.
$g_{1}^{U}$, $g_{2}^{U}$, $g_{3}^{U}$	Three ordered shared intermediates locally computed by party U.
*S*_A_, *S*_B_, *S*	Local and common session root keys.
KDF-SM3, *k*_s_	SM3-based key-derivation function and session-root output length.
*K*_c_, *k*_c_	Confirmation key and its output length.
*δ*_A_, *δ*_B_	Direction-distinguished HMAC-SM3 key-confirmation tags.
sid	Session identifier derived from hashed identities and deterministic encodings of *R*_A_ and *R*_B_.
label_i_, n_i_	Public purpose/direction label and output length of subkey i.
$K_{\mathrm{enc}}^{A\to B}$, $K_{\mathrm{mac}}^{A\to B}$	A-to-B encryption and authentication subkeys.
$K_{\mathrm{enc}}^{B\to A}$, $K_{\mathrm{mac}}^{B\to A}$	B-to-A encryption and authentication subkeys.
*type*, *seq*, *dir*, *len*	PDU type, sequence number, direction bit, and plaintext length.
*nonce*	SM4-CTR initialization value; it must be unique for a given directional encryption key.
*header*, *ciphertext*, τ	Authenticated PDU header, encrypted payload, and transmitted authentication tag; τ is distinct from *tag*_ID_.
ST[*ID*_B_]	T-Box session-state entry indexed by target ECU identity.
fresh, lifetime	Receiver freshness state and session-expiration bound.
T_KG_, T_AUTH_, T_DATA_	Offline key-generation, online authentication, and established-session data-plane times.

**Table 3:** ProVerif 2.05 query result summary.

Query	Verified Property	Result	Interpretation Within the Symbolic Model
Q1	Injective authentication of ECU B to T-Box A	True	Each T-Box acceptance corresponds to one matching ECU begin event.
Q2	Injective authentication of T-Box A to ECU B	True	Each ECU acceptance corresponds to one matching T-Box begin event.
Q3	Session-root key non-derivability	True	The modeled attacker cannot derive the root-key probe.
Q4	A → B encryption-subkey non-derivability	True	$K_{\mathrm{enc}}^{A\to B}$ remains unavailable to the modeled attacker.
Q5	A → B authentication-subkey non-derivability	True	$K_{\mathrm{mac}}^{A\to B}$ remains unavailable to the modeled attacker.
Q6	B → A encryption-subkey non-derivability	True	$K_{\mathrm{enc}}^{B\to A}$ remains unavailable to the modeled attacker.
Q7	B → A authentication-subkey non-derivability	True	$K_{\mathrm{mac}}^{B\to A}$ remains unavailable to the modeled attacker.
Q8	A → B service-data confidentiality	True	The A → B private service payload is non-derivable.
Q9	B → A service-data confidentiality	True	The B → A private service payload is non-derivable.
Q10	A → B integrity, origin, and one-time acceptance	True	Every accepted PDU matches one unique authentic send event.
Q11	B-to-A integrity, origin, and one-time acceptance	True	Every accepted PDU matches one unique authentic send event.

**Table 4:** Long-term-key and session-secret leakage boundary.

Reveal Condition	Newly Computable Information	Boundary Assessment	Permitted Claim
Passive transcript; later disclosure of *D*_A_ or *D*_B_	At most one directly cancellable pairing term	$g^{r_{A}r_{B}}$ remains behind the stated CDH assumption	Conditional passive/weak forward secrecy
Passive transcript; later disclosure of both identity keys or KGC master secret	$g^{r_{A}}$ and $g^{r_{B}}$	Past passive root still requires the CDH target; future node impersonation is possible	Not KGC-compromise resistance
*D*_U_ plus the matching *r*_U_ for the target session	The product term and session root can be reconstructed	Target session is exposed	Excluded by CK/eCK freshness
Current session-root or data-plane subkey disclosure	Current-session confidentiality/authentication material	No ratchet recovers the compromised session	No post-compromise security claim
Active long-term compromise during the target run or failure to erase ephemerals	Depends on reveal timing and retained process memory	Secure erasure is not guaranteed by the Python prototype	Outside the demonstrated boundary

**Table 5:** Single-ECU UDP handshake latency breakdown.

Metric	Value (ms)
T-Box completion time (median)	287.560
T-Box completion time (mean)	288.062
Standard deviation	2.866
T-Box ephemeral generation	32.509
T-Box SM9 session-key computation	201.473
T-Box subkey derivation	5.619
T-Box confirmation generation and verification	7.494
Link-establishment success rate	100%

**Table 6:** Host scalability measurements for 1–18 ECUs (three runs per point).

ECUs	*T*_KG_ Median (P95) (ms)	*T*_AUTH_ Median (P95) (ms)	*T*_AUTH_ per ECU (ms)	*T*_KC_ Total (ms)
1	215.529 (218.081)	496.099 (500.211)	496.099	14.958
4	248.699 (251.776)	1936.149 (1976.127)	484.037	58.126
8	296.091 (299.398)	3863.121 (3943.985)	482.890	117.130
12	341.182 (355.876)	5686.747 (5713.873)	473.896	172.113
18	405.416 (423.277)	8551.829 (8591.867)	475.102	256.859

**Table 7:** Data-plane performance across batch-size and tag-length configurations.

Profile	Batch Size/Tag (bit)	Frames/Message	Sender Median (ms)	Receiver Median (ms)	Total Median (P95) (ms)
Compact immediate	1/64	2.000	1.554	1.553	3.108 (3.163)
Compact batch	4/64	1.250	0.645	0.643	1.288 (1.303)
Compact batch	8/64	1.125	0.522	0.516	1.038 (1.055)
Recommended	8/128	1.250	0.521	0.516	1.036 (1.074)

**Table 8:** Cross-scheme communication cost under the common 8-B model.

Scheme/Profile	Bits/Message	Frames/Message	1 ECU	4 ECUs	18 ECUs	Evidence/Condition
No-security CAN baseline	111.00	1.00	2.220%	8.88%	39.96%	Common baseline
WSP [34]	131.00	1.00	2.620%	10.48%	47.16%	Literature lower bound
LEAP [32]	174.00	2.00	3.480%	13.92%	62.64%	Unified 8-B projection
EAS [35]	205.00	3.00	4.100%	16.40%	73.80%	Literature lower bound
MAuth-CAN [31]	222.00	2.00	4.440%	17.76%	79.92%	Bounded projection
Sensors 2025 [36]	166.00	1.50	3.320%	13.32%	59.94%	Data-dependent bound
Ours	138.75	1.25	2.775%	11.10%	49.95%	Repository deterministic mapping

**Table 9:** Life-cycle time decomposition and measurement boundaries.

Boundary	Symbol/Formula	Median (ms)	Payload/Scale	Measurement Boundary
offline key generation	*T*_KG_	204.851	T-Box + 1 ECU	10-run host life cycle
online authentication	*T*_AUTH_	475.163	1 ECU	Both endpoints, sequential
sender	*T*_S,opt_	0.521	8 B	30 independent blocks
receiver	*T*_R,opt_	0.516	8 B	30 independent blocks
data total	*T*_DATA,opt_	1.036	8 B	Paired per-block median
cold reference	*T*_KG_ + *T*_AUTH_ + *T*_S,opt_ + *T*_R,opt_	681.050	mixed runs	Estimate only; sum of marginal medians
online reference	*T*_AUTH_ + *T*_S,opt_ + *T*_R,opt_	476.199	mixed runs	Estimate only; sum of marginal medians

**Table 10:** Receiver outcomes for 12 representative frame-mutation attacks.

Attack Class	Representative Mutation	Rejected	Rejection Rate	Median/P95 (ms)	Plaintext-Release Trials
Ciphertext bit flip	D4 byte 1 XOR 0x01	100/100	100.0%	1.4184/1.5282	0
Authentication fragment 1 bit flip	A1 byte 1 XOR 0x80	100/100	100.0%	1.4120/1.4970	0
Authentication fragment 2 bit flip	A2 byte 8 XOR 0x01	100/100	100.0%	1.4228/1.5076	0
Zero-tag forgery	A1 = A2 = 0x00…00	100/100	100.0%	1.4042/1.4759	0
Data-frame deletion	Delete D5	100/100	100.0%	1.1837/1.2433	0
Data-frame insertion	Duplicate D6 before D6	100/100	100.0%	0.0037/0.0060	0
Data-frame reordering	Swap D2 and D7	100/100	100.0%	1.4190/1.4900	0
Full-batch replay	Replay accepted D1-D8, A1, A2	100/100	100.0%	1.4174/1.4692	0
Unknown CAN identifier	D1 ID: 321 -> 400	100/100	100.0%	0.0021/0.0039	0
Data/authentication identifier confusion	D3 ID: 321 -> 322	100/100	100.0%	0.0030/0.0051	0
Authentication-fragment truncation	Delete A2; detect at timeout	100/100	100.0%	0.0032/0.0044	0
Tag-profile downgrade	128-bit sender parsed as 64-bit profile	100/100	100.0%	1.4387/1.5006	0

**Table 11:** Loss detection and authenticated counter resynchronization.

Loss Scenario	Trials	Detection Rate	Damaged Plaintext Released	Recovery Rate	Recovery Frames/Bits	Median/P95 (ms)
Loss of D1	30	100.0%	0	100.0%	3/333	2.3270/2.4778
Loss of D4	30	100.0%	0	100.0%	3/333	2.3685/2.4583
Loss of D8	30	100.0%	0	100.0%	3/333	2.3697/2.4609
Loss of A1	30	100.0%	0	100.0%	3/333	2.4057/2.4710
Loss of A2	30	100.0%	0	100.0%	3/333	2.3663/2.4654
Loss of two data frames	30	100.0%	0	100.0%	3/333	2.3640/2.4331

**Table 12:** Qualified peer comparison of the proposed compact data plane.

Dimension	Proposed Profile	Peer Reference	Relative Result	Qualification
Wire cost (8-B message)	138.75 bits	WSP: 131 bits	5.92% higher	128-bit batch tag; standard CAN fields
Wire cost (8-B message)	138.75 bits	LEAP: 174 bits	20.26% lower	Unified 8-B projection
Wire cost (8-B message)	138.75 bits	EAS: 205 bits	32.32% lower	Authentication-only lower bound
Wire cost (8-B message)	138.75 bits	MAuth-CAN: 158-222 bits	12.18–37.50% lower	Trigger DLC is implementation dependent
18-ECU bus load	49.95%	Sensors 2025: 39.96–79.92%	Within range	Compression-dependent bound
Data-plane host time	1.036 ms/8 B	EC-SVC 0.753 ms/48 B; LEAP 1.998 ms/message	Not ranked	Hardware, payload, bus, and timing boundaries differ
Authentication strength	128-bit tag per 8-message batch	WSP: 32-bit tag	Longer tag; coarser granularity	Up to about 80 ms batch window at 100 Hz
Loss recovery	3 authenticated frames; 333-bit lower bound	No aligned peer result	Not ranked	Exceptional path; 0.666 ms wire-time lower bound
Classical-CAN compatibility	Standard IDs, CRC, and DLC8 payloads	WSP field repurposing	Controller-compatible mapping	Physical-controller validation pending

**Table 13:** Security functions and deployment boundaries.

Scheme	Confidentiality	Integrity/Replay	Route/Access	CAN Mapping	DoS/Bus-Off	Main Limitation
Ours	Yes	Yes/tested	Tested/policy binding	Standard	Limited/not tested	batch delay, tag truncation, and resynchronization
WSP [34]	Yes	Yes/yes	Threat-model limited/no	CRC/extended-ID repurposing	Not central/no	tested controller could not modify those fields
Lightweight CAN [37]	Yes	Yes/yes	Yes/no	CRC/extended-ID repurposing	Not central/no	32-bit tag and nonstandard field use
EAS [35]	No	Yes/yes	Yes/no	RTR-dependent	Not central/no	authentication-only and extra RTR traffic
MAuth-CAN [31]	No	Yes/yes	Yes/no	Standard controller	Partial/yes	authentication-only; trigger/wait overhead
LEAP [32]	Yes	Embedded ID/tested	Tested/pairwise	Application repacking	Tested/no	RC4 design and only 11 identity bits
EC-SVC [30]	Yes	Yes/yes	Access policy/yes	CAN-FD	Not central/no	edge dependency and expensive access-control setup
Sensors 2025 [36]	Tested	Tested/tested	Brute-force test/no	Compression dependent	Tested/no	load advantage depends on trace compressibility

**Table 14:** Load, schedulability, and authentication-release bounds at 18 ECUs, 100 Hz, and a 128-bit tag.

K	Unstuffed Load	Stuffed Upper	Bus Response (ms)	First (ms)	Average (ms)	Last (ms)	Status
1	119.88%	145.80%	No finite deadline	--	--	--	Not schedulable
2	79.92%	97.20%	19.44	33.7599	28.7599	23.7599	Capacity only
4	59.94%	72.90%	29.16	64.8053	49.8053	34.8053	Capacity only
8	49.95%	60.75%	48.60	127.4684	92.4684	57.4684	Capacity + 70% screen

**Table 15:** T-Box session-key operation profile.

Operation	Mean (ms)	Median (ms)	SD (ms)	P95 (ms)	Mean Share	Precomputation Boundary
Variable pairing e(R_peer_,D_self_)	177.461	177.036	4.041	183.674	86.41%	Online fresh R
Fixed-base exponentiation g^x^	10.491	10.405	0.463	11.138	5.11%	Current session/local
Pairing-result exponentiation $g_{1}^{x}$	10.570	10.477	0.491	11.571	5.15%	Current session/local
Encoding, hashes, and KDF	6.849	6.808	0.301	7.377	3.34%	Current session/local

**Table 16:** Multi-ECU establishment makespan across 30 runs per point.

ECUs	Sequential Median (P95), ms	Concurrent Median (P95), ms	Concurrent + Safe Precomputation Median (P95), ms	Parallel Speedup	Combined Reduction
1	297.360 (309.457)	299.313 (310.426)	260.340 (271.419)	0.993×	12.45%
4	1190.354 (1212.878)	1021.041 (1059.582)	950.500 (976.097)	1.166×	20.15%
8	2376.276 (2404.369)	2022.301 (2090.544)	1875.440 (1927.707)	1.175×	21.08%
12	3577.565 (3623.452)	3018.229 (3113.232)	2809.737 (2870.775)	1.185×	21.46%
18	5368.785 (5393.825)	4509.737 (4584.788)	4201.479 (4302.817)	1.190×	21.74%

## Data Availability

The data supporting the findings of this study are available from the corresponding author upon reasonable request. The data are not publicly available due to confidentiality restrictions associated with the industrial collaboration.

## Appendix A. Supplementary Evaluation Tables

This appendix provides detailed evidence classifications, analytical mappings, sensitivity results, and extended host-side measurements that support the summaries reported in the main text.

**Table A1:** Evidence categories for cross-study comparisons.

Class	Evidence Type	Examples in This Paper	Controlled Boundary	Permitted Use
S	Same-platform baseline	None for peer schemes	Same hardware, software, payload, traffic, and timing boundary required	No cross-scheme performance rank is supported because this class is empty
H	Host measurement	287.560 ms; 201.473 ms; 1.036 ms/8 B; host attack and recovery timings	Windows/Python/UDP or host harness; no physical bus or embedded controller	Describe prototype behavior only
A	Analytical model	138.75 bit/message; 49.95%; 60.75% sensitivity	500 kbit/s classical-CAN, 100 messages/s/ECU, stated frame-length assumptions	Offered-load and sensitivity analysis only
L	Literature report	Peer timings, formulas, payloads, and source-specific bounds	Original authors’ platform, algorithm, payload, and metric boundary	Engineering context; no direct rank
P	Unified or bounded projection	LEAP, EAS, MAuth-CAN, and Sensors mappings to a common 8-B view	Assumption-dependent remapping; no reproduced execution	Compare projected frame cost only

**Table A2:** Analytical communication-cost mapping and evidence sources.

Scheme or Profile	Evidence Class	Basis	Common-Model Value	Permitted Interpretation
No-security CAN baseline	A	One unstuffed standard frame per 8-B message	111.00 bits; 39.96% at 18 ECUs	Common analytical reference only
WSP [34]	L → P	131-bit literature lower bound mapped with the common load formula	47.16%	Lower-bound context; not reproduced
LEAP [32]	P	Unified 8-B projection from the cited design	174 bits; 62.64%	Projected traffic only
EAS [35]	L → P	205-bit authentication-only lower bound mapped with the common formula	73.80%	Different protected scope; no advantage claim
MAuth-CAN [31]	P	Bounded trigger and frame-format projection	158–222 bits; 56.88–79.92%	Projection range; upper endpoint is not a single measured result
Sensors 2025 [36]	L → P	Compression-dependent 1.50-frame reference point	166.50 bits; 59.94%	Trace-dependent point; the retained 166.00 value is arithmetically inconsistent
Proposed profile	A	Ten 111-bit frames for eight protected messages	138.75 bits; 49.95%	Deterministic unstuffed projection; no physical-bus measurement

**Table A3:** Timing components and reported reference points.

Scheme	Key Generation/Update	Authentication	Data Processing	Platform/Payload	Boundary Qualification
Ours	T_KG_ = 204.851 ms	T_AUTH_ = 475.163 ms	1.036 ms/8 B	Windows/Python host	sequential SM9; no bus time
Lightweight CAN [37]	41.630 ms generation; 2TM + Tv = 0.24 ms update	4TM = 0.45 ms	<378 µs at 80 MHz (original test)	Raspberry Pi 3B	32-bit tag; separate values
WSP [34]	4TM + 2TA = 20.45 ms	2TM + 2TA = 20.22 ms	<378 us at 80 MHz in original test	protocol-specific hardware	formula/hardware rows differ
EAS [35]	4TM = 0.45 ms	4TM + 2TA = 20.45 ms	authentication only	harmonized operation cost	two RTR frames
MAuth-CAN [31]	not reported	3TM + Tw = 0.34 ms + Tw	authentication only	CAN prototype	Tw unresolved
LEAP [32]	199.4 ms total update	merged with data path	1.998 ms/CAN message	ATmega328, 16 MHz	RC4; 11-bit embedded ID
EC-SVC [30]	424 ms at 16 ECUs	access-control/key phase	0.753 ms/48 B sharing	TMS320 + edge, CAN FD	includes communication in sharing
Sensors 2025 [36]	DBK 1.144/0.0858 ms	merged security module	0.85/0.064 ms	30/400 MHz	compression dependent

**Table A4:** Evidence categories and comparability of the timing data.

Scheme	Evidence Class	Value Retained	Platform or Payload Boundary	Use in This Paper
Proposed	H	T_KG_ 204.851 ms; T_AUTH_ 475.163 ms; 1.036 ms/8 B	Windows/Python host; sequential SM9; no bus time; separate benchmark blocks	Within-prototype decomposition only
Lightweight CAN [37]	L	41.630 ms generation; source formulas; <378 µs reported reference	Source-specific Raspberry Pi, 80-MHz, and tag boundaries are not one common run	Context only; source boundary must be consulted
WSP [34]	L/P	Operation formulas and <378 µs source reference	Protocol-specific hardware and formula boundaries	No direct timing comparison
EAS [35]	P	Harmonized operation-cost formulas	No common execution; authentication-only scope	Model reference only
MAuth-CAN [31]	L/P	0.34 ms plus unresolved Tw	CAN prototype; unresolved wait term	Incomplete reference; no rank
LEAP [32]	L	199.4 ms update; 1.998 ms/message	ATmega328, 16 MHz; RC4; source payload boundary	Source-reported context
EC-SVC [30]	L	424 ms at 16 ECUs; 0.753 ms/48 B	TMS320 plus edge node; CAN FD; includes communication in sharing	Source-reported context
Sensors 2025 [36]	L	DBK 1.144/0.0858 ms; 0.85/0.064 ms	30/400 MHz; compression-dependent design	Source-reported context

**Table A5:** Authenticated resynchronization under sustained message loss.

Lost Messages	Success/Trials	Recovery Rate	Median Resync Processing (ms)	P95 Resync Processing (ms)
1	5/5	100%	1.1244	1.3070
8	5/5	100%	1.3178	1.4136
64	5/5	100%	1.0983	1.3485
256	5/5	100%	1.1585	1.4960
1024	5/5	100%	1.1614	1.2076
4096	5/5	100%	1.1228	1.2497
16,384	5/5	100%	1.1359	1.2621

**Table A6:** Analytical cross-scheme communication-cost differences.

Dimension	Evidence Class	Comparable Statement	Non-Comparable Factors	Permitted Conclusion
Wire cost vs. WSP, LEAP, EAS, MAuth-CAN	P	Within the 8-B mapping, the arithmetic deltas are +5.92%, −20.26%, −32.32%, and −12.18% to −37.50%	Frame assumptions, tag strength, protected scope, triggers, and source platform differ	Projected frame-cost differences only
Sensors bus load	P	The proposed 49.95% point lies within the retained 39.96–79.92% compression-dependent range	Trace compressibility and framing assumptions differ	Range context only
Host time vs. peers	H vs. L	The numerical values may be reported with their own units and payloads	Hardware, payload, algorithm, software, and timing boundaries differ	No relative speed claim
Authentication strength	Qualitative	A 128-bit tag protects each eight-message batch; WSP reports a 32-bit tag	Tag length, aggregation granularity, and delay differ	Security and latency trade-off
Loss recovery	H/A	Three authenticated frames; 333-bit unstuffed lower bound	No aligned peer result and no physical-bus test	Implemented recovery path only
Classical-CAN compatibility	Qualitative	The mapping retains standard identifiers, CRC handling, and DLC-8 payloads	No physical controller interoperability test	Design compatibility pending validation

**Table A7:** Host processing time for complete authenticated batches.

k	Tag (Bit)	Batches	Batch P50 (ms)	Batch P95 (ms)	Per-Message P95 (ms)	Frames/Message
1	64	3200	3.0297	3.5903	3.5903	2.000
1	128	3200	3.0353	3.6125	3.6125	3.000
2	64	1600	3.7205	4.3113	2.1557	1.500
2	128	1600	3.7229	4.3199	2.1599	2.000
4	64	800	5.0802	5.6647	1.4162	1.250
4	128	800	5.0808	5.6453	1.4113	1.500
8	64	400	8.2383	8.9049	1.1131	1.125
8	128	400	8.2173	8.8684	1.1085	1.250

**Table A8:** Message-rate sensitivity for k = 8, 18 ECUs, and a 128-bit tag.

Rate (Hz)	Stuffed Load	First Accumulation (ms)	Bus Response (ms)	First Release (ms)	Average Release (ms)	Last Release (ms)
10	6.075%	700.0	48.6	757.4684	407.4684	57.4684
20	12.150%	350.0	48.6	407.4684	232.4684	57.4684
50	30.375%	140.0	48.6	197.4684	127.4684	57.4684
100	60.750%	70.0	48.6	127.4684	92.4684	57.4684

**Table A9:** Ideal online-forgery bounds for 18 ECUs at 100 Hz over ten years.

Tag (Bit)	Completed Batches	Per-Attempt Probability	Batch-Attempt Union Bound	Message-Equivalent Bound	Messages Exposed/Accepted Batch
64	71,004,600,000	5.4210 × 10^−20^	3.8492 × 10^−9^	3.0793 × 10^−8^	8
128	71,004,600,000	2.9387 × 10^−39^	2.0866 × 10^−28^	1.6693 × 10^−27^	8

**Table A10:** Per-ECU completion-time variation at 18 ECUs.

Mode	Total Median (ms)	Total/18 Median (ms)	Observed Session Median (ms)	Session SD (ms)	Session P95 (ms)
Sequential	5368.785	298.266	297.298	6.360	309.341
Concurrent	4509.737	250.541	4176.826	168.594	4446.793
Concurrent + safe precomputation	4201.479	233.415	3927.996	134.614	4147.141

**Table A11:** Representative access patterns.

Access Pattern	ECUs	Concurrent Median (ms)	Safe Precomputation Median (ms)	Operational Interpretation
On-demand remote diagnostic	1	299.313	260.340	one explicitly requested ECU
Small functional-domain diagnostic	4	1021.041	950.500	four ECUs in one subsystem
Staged OTA cohort	8	2022.301	1875.440	first deployment cohort
Expanded OTA cohort	12	3018.229	2809.737	larger staged deployment
Synchronized startup or expiry recovery	18	4509.737	4201.479	upper tested gateway burst
